# Relationship between vaginal and oral microbiome in patients of human papillomavirus (HPV) infection and cervical cancer

**DOI:** 10.1186/s12967-024-05124-8

**Published:** 2024-04-29

**Authors:** Wei Zhang, Yanfei Yin, Yisha Jiang, Yangyang Yang, Wentao Wang, Xiaoya Wang, Yan Ge, Bin Liu, Lihe Yao

**Affiliations:** 1 The First School of Clinical Medicine, Lanzhou University, Lanzhou, China; 2 School/Hospital of Stomatology, Lanzhou University, Lanzhou, China; 3 Healthy Examination & Management Center of Lanzhou University Second Hospital, Lanzhou, China; 4 Department of Neurology, Lanzhou University First Hospital, Lanzhou, China; 5 Department of Gynecology, Lanzhou University First Hospital, Lanzhou, China

**Keywords:** Vaginal microbiome (VM), Human papillomavirus (HPV), Cervical cancer, Oral microbiome, 16S rRNA

## Abstract

**Background:**

The aim of this study was to assess the microbial variations and biomarkers in the vaginal and oral environments of patients with human papillomavirus (HPV) and cervical cancer (CC) and to develop novel prediction models.

**Materials and methods:**

This study included 164 samples collected from both the vaginal tract and oral subgingival plaque of 82 women. The participants were divided into four distinct groups based on their vaginal and oral samples: the control group (Z/KZ, n = 22), abortion group (AB/KAB, n = 17), HPV-infected group (HP/KHP, n = 21), and cervical cancer group (CC/KCC, n = 22). Microbiota analysis was conducted using full-length 16S rDNA gene sequencing with the PacBio platform.

**Results:**

The vaginal bacterial community in the Z and AB groups exhibited a relatively simple structure predominantly dominated by *Lactobacillus.* However, CC group shows high abundances of anaerobic bacteria and alpha diversity. Biomarkers such as Bacteroides, Mycoplasma, Bacillus, *Dialister*, *Porphyromonas*, *Anaerococcus*, and *Prevotella* were identified as indicators of CC. Correlations were established between elevated blood C-reactive protein (CRP) levels and local/systemic inflammation, pregnancy, childbirth, and abortion, which contribute to unevenness in the vaginal microenvironment. The altered microbial diversity in the CC group was confirmed by amino acid metabolism. Oral microbial diversity exhibited an inverse pattern to that of the vaginal microbiome, indicating a unique relationship. The microbial diversity of the KCC group was significantly lower than that of the KZ group, indicating a link between oral health and cancer development. Several microbes, including *Fusobacterium*, *Campylobacter*, *Capnocytophaga*, *Veillonella*, *Streptococcus*, *Lachnoanaerobaculum*, *Propionibacterium*, *Prevotella*, *Lactobacillus*, and *Neisseria,* were identified as CC biomarkers. Moreover, periodontal pathogens were associated with blood CRP levels and oral hygiene conditions. Elevated oral microbial amino acid metabolism in the CC group was closely linked to the presence of pathogens. Positive correlations indicated a synergistic relationship between vaginal and oral bacteria.

**Conclusion:**

HPV infection and CC impact both the vaginal and oral microenvironments, affecting systemic metabolism and the synergy between bacteria. This suggests that the use of oral flora markers is a potential screening tool for the diagnosis of CC.

**Supplementary Information:**

The online version contains supplementary material available at 10.1186/s12967-024-05124-8.

## Introduction

Cervical cancer (CC) ranks as the fourth most frequently diagnosed cancer and the third leading cause of cancer death in the women globally. More than half a million CC cases are annually linked to (human papillomavirus) HPV infection, resulting in 250,000 deaths for per year [1]. HPV infection is recognized as one of the major causes of CC, as well as other malignancies including anogenital tumors, anal, vulvar, penile, vaginal and oral cancers [2, 3]. Recent studies suggest that 90% of oral squamous cell carcinomas are caused by HPV infections [4].

The oral cavity is a natural open system composed of a complex microbiome consisting of more than 800 bacterial species [5]. This oral microbiota has been implicated not only in periodontal disease but also in systemic conditions including haematological diseases, and lymphatic, lung, pancreatic and breast cancers [6–10].

Changes in the composition of the oral microbiome are now recognized as potential biomarkers for cancers*,* including colorectal cancer (CRC)*,* marked by increased *Fusobacterium nucleatum* abundance [11, 12]. Similarly, shifts favouring oral pathogens such as the genera *Porphyromonas*, *Fusobacterium* and *Prevotella* have been correlated with the incidence of CC, although the underlying mechanisms remain poorly understood [13], these oral pathogens also cause the periodontal disease [14]. Previous studies have shown a correlation between vaginal bacteria and gingival inflammation [15], HPV not only invades the basal cells of the vaginal epithelium, but also infects the periodontal tissue and keeps the virus in a latent state [5]. The oral and vaginal environments may provide similar colonization and growth conditions, resulting in a significantly increased risk of periodontal disease and cancer [16]. However, relatively few studies exist on changes in the oral microbiome when the vaginal microbiota is transformed during the course of HPV infection to CC development, suggesting that more research is needed in this area.

Population-based CC screening is implemented as a public health priority in China [17], and the best strategy is the use of a liquid-based cytology test (TCT) combined with HPV screening [18, 19]. The combined screening technology, although advanced and effective, is costly and suitable for areas with adequate medical and health care. However, there are still many less economically developed areas (rural areas) in China, and there is an urgent need to find a high-quality and inexpensive method. Changes in oral microbial diversity that are detectable through low-cost profiling may offer additional value as biomarkers for early screening, diagnosis and monitoring of HPV infection and even CC. The aim of this study was to evaluate the differences and associations between vaginal and oral microorganisms in HPV-infected patients and CC patients. An increased understanding of microbial ecology may contribute to improving the accuracy of CC screening and providing life-saving interventions to vulnerable groups.

## Materials and methods

### Study design and specimen collection

Ethical approval was obtained from the Ethics Committee of the Second Hospital of Lanzhou University (approval no. 2022A-533). All methods were conducted in accordance with the Declaration of Helsinki and relevant guidelines and regulations. Participants were recruited from October 2022 to October 2023 at outpatient and inpatient gynaecology departments.

The inclusion criteria were as follows: (1) aged 20–76 years, sexually active; (2) no vaginal irrigation or antibiotic treatment for ≥ 1 week before the examination and no sexual intercourse within 3 days; (3) had regular menstrual cycles; (4) had no history of cervical surgery or hysterectomy and no diseases seriously affecting other systems; (5) were positive for HPV infection by diagnostic testing; (6) had CC confirmed by biopsy tissue histopathological examination; and (7) had undergone abortion via surgery and had ≥ 3 abortions. The exclusion criteria were as follows: (1) menstruation; (2) vaginal bleeding; (3) intrauterine device use; (4) pregnancy or lactation; (5) radiation and chemotherapy; and (6) prior medical or spontaneous abortion.

All participants completed a clinical questionnaire, oral examination results were recorded, and had specimen and blood samples collected. Caries were diagnosed using the diagnostic criteria [20] of the International Caries Detection and Assessment System (ICDAS) [21]. Periodontitis was defined as ≥ 4 teeth with ≥ 1 site having a probing depth ≥ 4 mm, clinical attachment loss ≥ 1 mm and bleeding upon probing. For subgingival plaque sample collection, the subjects could not eat, drink, smoke or chew gum, and the debris in their mouth was washed with drinking water before 30 min. The sterile dental scaler penetrated the tongue side of the mandibular first molar below the gum to remove dental plaque and blood contamination was prevented during the collection process. For vaginal secretion sample collection, a disposable speculum was inserted, and a sterile swab sample was taken from the posterior vaginal fornix. All specimens were stored immediately at − 80 °C for DNA extraction. Blood indicators and C-reactive protein (CRP) levels were analysed using an automated blood analyser system (SysmexXN-20; Kobe, Japan).

### Full-length 16S sequencing and data processing

Bacterial genomic DNA was extracted from vaginal and subgingival samples using the TGuide S96 Magnetic Universal DNA Kit (Tiangen Biotech (Beijing) Co., Ltd.) according to the manufacturer’s instructions. The full-length 16S rRNA gene was amplified with the primer pair 27F (AGRGTTTGATYNTGGCTCAG) and 1492R (TASGGHTACCTTGTTASGACTT). Both the forward and reverse 16S primers were tailed with sample-specific PacBio barcode sequences to allow for multiplexed sequencing. After the individual quantification step, amplicons were pooled in equal amounts. SMRTbell libraries were prepared from the amplified DNA with the SMRTbell Express Template Prep Kit 2.0 according to the manufacturer’s instructions (Pacific Biosciences). Purified SMRTbell libraries from the pooled and barcoded samples were sequenced on a PacBio Sequel II platform (Beijing Biomarker Technologies Co., Ltd., Beijing, China) using a Sequel II binding kit 2.0. The raw subreads were corrected by circular consensus sequencing (CCS) (SMRT Link, version 8.0), and then, Lima software (v1.7.0) was used to identify different CCS samples by barcode. Cutadapt software (1.9.1) was used to identify and remove the primer sequences via length filtering, and the chimeric sequences were identified and removed by UCHIME (version 8.1). A high-quality CCS sequence was obtained.

### Statistical analysis

Demographic and oral data and blood indices were compared between groups using SPSS 27.0 (IBM, Chicago, Illinois, USA). Comparisons between groups were made by analysis of variance (ANOVA), Fisher's precision probability test and the chi-square test. The online platform BMKCloud (https://www.biocloud.net) was used to analyse the sequencing data. R (v3.2.0) was used to construct Venn and bubble diagrams for correlation analysis. Analysis of alpha diversity was performed to determine the complexity of the species diversity of each sample utilizing QIIME2 software. Beta diversity among the samples was evaluated by principal coordinate analysis (PCoA) to assess the diversity of the samples for species complexity. One-way ANOVA was used to compare bacterial abundance and diversity. Linear discriminant analysis (LDA) coupled with effect size (LEfSe) was applied to evaluate the differentially abundant taxa. An LDA score > 2 was considered the cut-off value for biomarker screening, and the differences between groups were assessed by the rank sum test. At the genus level, a threshold p value of < 0.05 and SparCC were used to construct a correlation plot. Random forest analysis was performed to compare the microbiome characteristics, and the area under the receiver operating characteristic curve (AUC) was assessed to evaluate the performance of the random forest analysis. Statistical comparison of AUCs was performed using the pROC package in R. Picrust2 was used for Kyoto Encyclopedia of Genes and Genomes (KEGG) functional prediction.

## Results

### Characteristics of the subjects

The participants included 82 women, and 164 vaginal and gingival samples were sequenced. The patients were divided into four groups: control group: the vaginal secretion (Z) and oral subgingival plaque (KZ), abortion group (AB/KAB): HPV genotype test and TCT were negative, abortion was performed by surgical method, and the number of abortions was ≥ 3. In the HPV group (HP/KHP), the HPV test results were positive, the TCT results were normal, and in the CC/KCC group, the HPV test results were positive and confirmed by cervical biopsy. The demographic, clinical, and oral health parameters are shown in Table 1.

**Table 1:** Demographic information, blood indices, oral characteristics and different HPV distribution of participants

	Normal (n = 22)	Abortion (n = 17)	HPV (n = 21)	Cancer (n = 22)	P value
Age (year)	44.68 ± 8.54	43.12 ± 7.67	45.76 ± 11.02	48.05 ± 8.99	0.395
Nation					
Han nationality	22 (100)	17 (100)	21 (100)	17 (77.27)	**0.003**
Other nationalist	0 (0)	0 (0)	0 (0)	5 (22.73)	
Level of education					
Primary school	1 (4.55)	6 (35.29)	13 (61.90)	19 (86.36)	**< 0.001**
High school	8 (36.36)	6 (35.29)	3 (14.29)	2 (9.09)	
College degree	13 (59.09)	5 (29.41)	5 (23.81)	1 (4.55)	
Gravidity	0.87 ± 0.94	4.82 ± 1.13	2.48 ± 1.50	3.14 ± 1.61	**< 0.01**
Surgical abortions	0.32 ± 0.48	3.29 ± 0.59	0.62 ± 0.74	0.59 ± 0.85	**< 0.01**
Parity	0.55 ± 0.74	1.53 ± 0.94	1.86 ± 1.28	2.55 ± 1.34	**< 0.01**
Sexual age (year)	21.18 ± 2.70	21.59 ± 3.47	21.81 ± 3.82	20.45 ± 3.10	0.556
BMI (kg/m^2^)	20.60 ± 1.90	23.61 ± 4.98	22.31 ± 3.03	23.63 ± 3.89	**0.02**
Smoking	2 (9.09)	3 (17.65)	0 (0)	0 (0)	0.059
Drinking	4 (18.19)	6 (35.30)	3 (14.29)	4 (18.19)	0.481
Family history of CC (%)	1 (4.54)	2 (11.76)	2 (9.52)	0 (0)	0.365
CRP (mg/dL)	0.31 ± 0.31	1.89 ± 1.45	2.23 ± 1.79	1.62 ± 1.00	**< 0.01**
WBC (4.5 to 11.0 × 10^9^/L)	7.68 ± 3.12	7.75 ± 1.78	6.15 ± 1.88	6.82 ± 2.80	0.635
RBC	4.60 ± 0.36	4.54 ± 0.44	4.46 ± 0.38	4.38 ± 0.57	0.068
HGB	132.41 ± 16.98	135.06 ± 11.14	126.95 ± 15.45	125.55 ± 22.49	0.122
NEUT%	65.42 ± 10.75	64.91 ± 7.76	59.7 ± 7.07	70.20 ± 11.03	**0.014**
LYM%	27.88 ± 9.69	28.21 ± 7.43	33.02 ± 7.57	23.66 ± 9.83	0.079
MONO%	5.30 ± 1.09	5.63 ± 1.05	5.17 ± 1.29	4.75 ± 1.45	0.461
EOS%	1.06 ± 0.91	0.94 ± 0.63	1.70 ± 2.02	0.93 ± 0.70	**0.048**
BASO%	0.34 ± 0.30	0.26 ± 0.16	0.41 ± 0.28	0.45 ± 0.33	0.118
PLT	288.09 ± 69.22	266.59 ± 51.26	256.76 ± 71.33	240.55 ± 88.44	0.158
Caries (%)	3 (13.64)	2 (11.76)	1 (4.76)	2 (9.09)	0.663
Periodontitis (%)	3 (13.64)	3 (17.65)	5 (23.81)	8 (36.36)	0.34
Frequency of tooth brushing (%)					
≤ 1times/day	3 (13.64)	8 (47.06)	8 (38.10)	16 (72.73)	**< 0.001**
2 times/day	19 (86.36)	9 (52.94)	13 (61.90)	6 (27.27)	
Brushing time (%)					
≤ 1 min	1 (4.55)	3 (17.65)	12 (57.14)	9 (40.91)	**< 0.001**
2 min	7 (31.82)	7 (41.18)	6 (28.57)	11 (50.00)	
≥ 3 min	14 (63.66)	7 (41.18)	3 (14.29)	2 (9.09)	
Gingival bleeding (%)					
No	16 (72.73)	9 (52.94)	7 (33.33)	15 (68.18)	**0.043**
Yes	6 (27.27)	8 (47.06)	14 (66.67)	7 (31.82)	
Professional dental cleaning (%)					
No	16 (72.73)	12 (70.59)	18 (85.71)	22 (100)	**0.039**
Once a year	5 (22.73)	2 (11.76)	3 (14.29)	0	
2–4 years/times	0	1 (5.89)	0	0	
≥ 5 years/times	1 (4.55)	2 (11.76)	0	0	
Toothache or sensitivity of tooth (%)					
No	20 (90.91)	15 (88.24)	17 (80.95)	17 (77.27)	0.768
Yes	2 (9.09)	2 (11.76)	4 (19.05)	5 (22.73)	
HPV subtypes					
16	–	–	13 (61.90)	12 (54.55)	–
18	–	–	4 (19.05)	6 (27.27)	
33	–	–	1 (4.76)	1 (4.55)	
51	–	–	1 (4.76)	1 (4.55)	
52	–	–	1 (4.76)	0	
53	–	–	1 (4.76)	2 (9.09)	
58	–	–	3 (14.29)	5 (22.73)	

*BMI* body mass index, *CRP* C-reactive protein, *WBC* White Blood Cell Count, *RBC* Red Blood Count, *HGB* hemoglobin, *NEUT%* neutrophilic granulocyte percentage, *LYM%* lymphocytes percentage, *MONO%* monocytes percentage, *EOS%* eosinophils percentage, *BASO%* basophil percentage, *PLT* Platelets

The P-value is calculated by ANOVA, Fisher exact probability test. Bold value indicates that P value is less than 0.05

There was no significant difference among the three groups regarding age, sex, smoking status, drinking status, or family history of CC (all *p* ≥ 0.05). At the national level of education, number of pregnancies, number of abortions, number of childbirths, and BMI were significantly different (p values ranged from 0.01–0.001). The systemic inflammation marker CRP and eosinophil percentage (EOS%) were greater in patients with HPV + infection, and the neutrophilic granulocyte percentage (NEUT%) was greater in patients with CC than in their respective controls. Dental caries and periodontal disease were not significantly different between the groups. However, there were significant differences in oral hygiene practices, including brushing frequency and duration, gingival bleeding and professional cleaning. HPV genotype distribution among the infected groups revealed a predominance of 56%, 23%, and 19% high-risk HPV16, 18 and 58 strains, respectively. Other high-risk HPV genotypes, including 33, 51, 52, 53, and 68, and some patients were infected with multiple genotypes of HPV, accounted for 23.26% of infections (Table 1).

### Composition and diversity of vaginal and oral microbiota

**Fig. 1:**
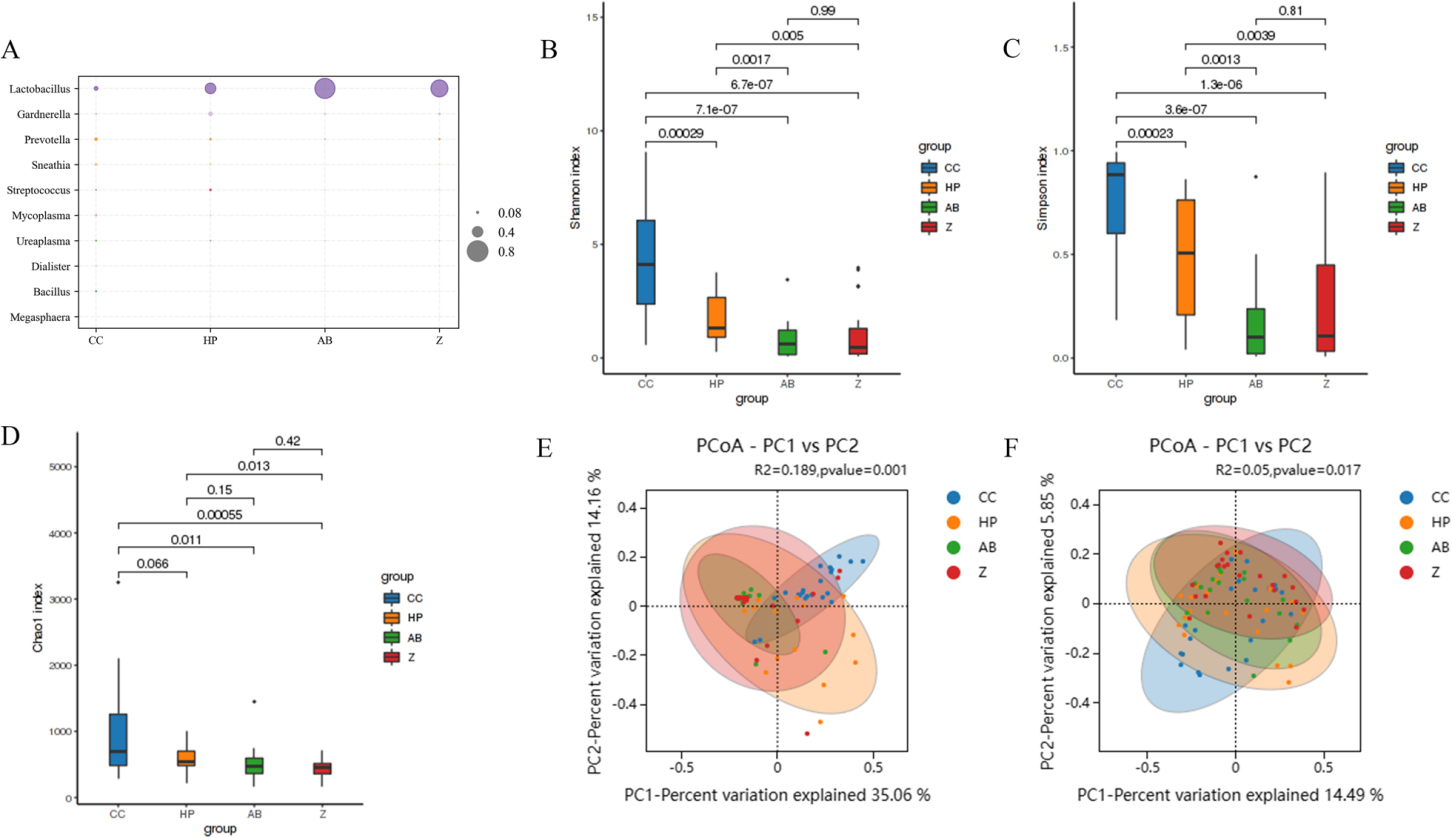
Community compositions and diversity at the genus level of vaginal microbes. **A** Bubble diagram of relative abundance of top ten genus of each group, the size of the dots represents the species proportion. Microbiota Alpha diversity; **B** Shannon index; **C** Simpson index; **D** Chao 1 index. Principal coordinate analysis (PCoA) derived from weighted (**E**) and unweighted (**F**) UniFrac distances among the samples of the four groups. Every group is represented by diferent colors

Venn diagrams showed the highest number of unique OTUs in the CC vaginal microbiota, while the control (Z) and AB groups exhibited substantial overlap (Additional file 1: Figure S1A). A total of 736 OTUs were shared among the groups. Additional file 1: Figure S1C shows the composition of bacteria greater than 0.1% at the genus level in vaginal specimens. The top ten bacterial species in terms of relative abundance are shown on the bubble plot (Fig. 1A). *Lactobacillus* was dominant among the AB group (88.45%) and controls (74.75%), and its abundance declined drastically in the CC samples (16.24%). Alpha diversity metrics, including the Shannon, Simpson and Chao1 indices, significantly differed according to vaginal microbiome richness and evenness (all *p* < 0.05) (Fig. 1B–D). We confirmed that the composition of the vaginal microbiota gradually changed from the control group to the HPV infection group and then to the CC group, which had the highest alpha diversity (*p* < 0.05). PCoA based on weighted and unweighted UniFrac distances was also conducted. According to the weighted UniFrac analysis, the first and second primary components accounted for 35.06% and 14.16%, respectively, while according to the unweighted UniFrac analysis, the first and second primary components accounted for 14.49% and 5.85%, respectively (Fig. 1E, F). The community population distributions of the four groups overlapped; however, the weighted and unweighted UniFrac PERMANOVA suggested that the microbiota distribution varied significantly among the groups (*p* = 0.001, *p* = 0.017).

**Fig. 2:**
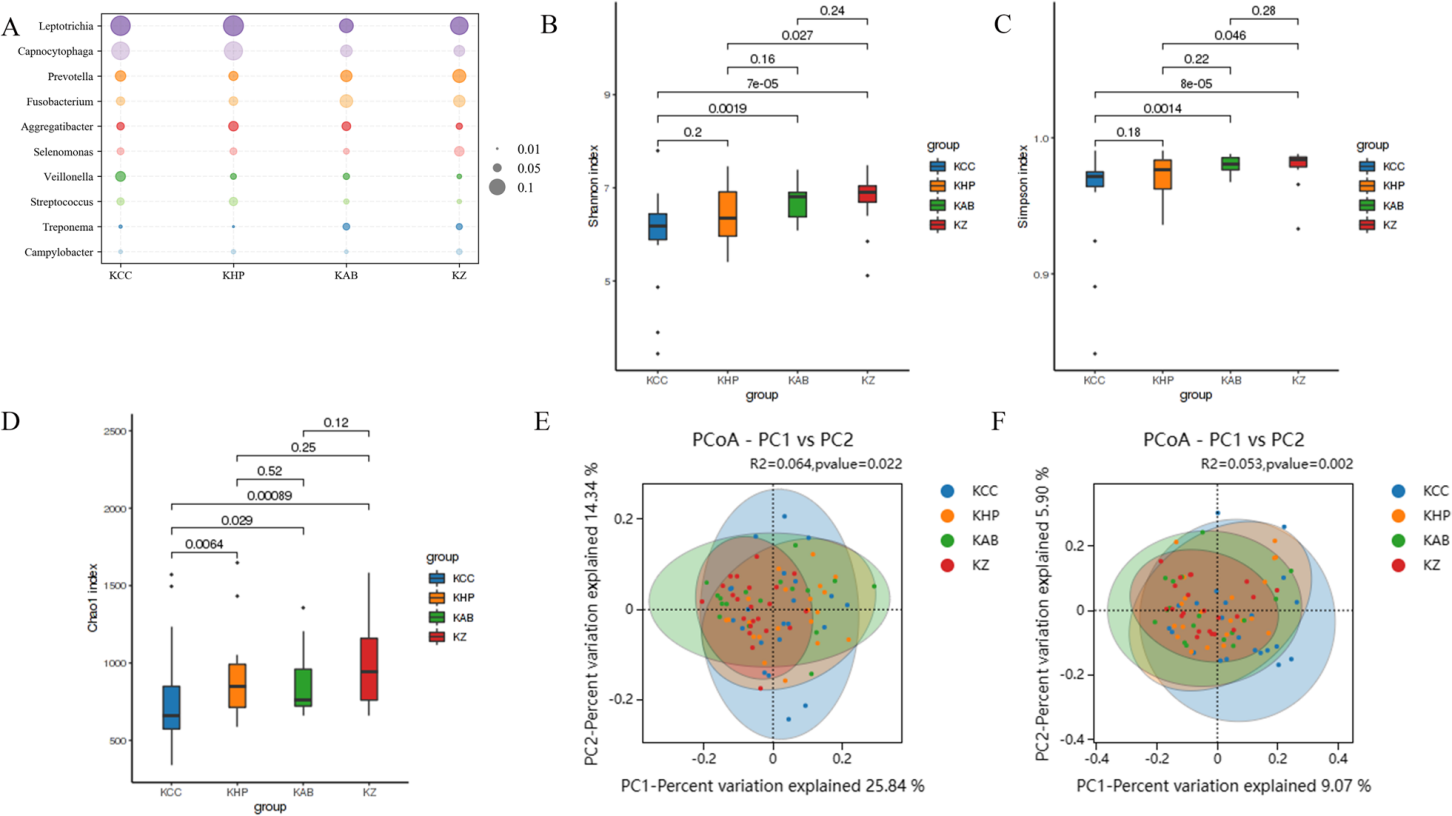
Community compositions and diversity at the genus level of oral microbes. **A** Bubble diagram of relative abundance of top ten genus of each group, the size of the dots represents the species proportion. Microbiota Alpha diversity; **B** Shannon index; **C** Simpson index; **D** Chao 1 index. Principal coordinate analysis (PCoA) derived from weighted (**E**) and unweighted (**F**) UniFrac distances among the samples of the four groups. Every group is represented by diferent colors

In the oral microbiota, there was no significant difference in the number of OTUs among the four groups (Additional file 1: Figure S1B), and a total of 1411 OTUs overlapped. There were significantly more oral microorganisms than vaginal microorganisms in terms of species number and abundance (Additional file 1: Figure S1D). The top ten most abundant species of oral microorganisms were *Leptotrichia*, *Capnocytophaga*, *Prevotella*, *Fusobacterium*, *Aggregatibacter*, *Selenomonas*, *Veillonella*, *Streptococcus*, *Treponema*, and *Campylobacter* (Fig. 2A). The KZZ and KCC groups showed the most significant differences in alpha diversity (for the Shannon index, Simpson index, and Chao1 index, values of *p* < 0.05 were noted) (Fig. 2B–D). The oral microbiome diversity was highest in the control group and lowest in the CC group, in contrast to changes in the vaginal microbiome. Weighted and unweighted UniFrac PERMANOVA also revealed that the beta diversity of the oral microbiota varied significantly among the groups (*p* = 0.022, *p* = 0.002) (Fig. 2E, F).

### Identification of Z and CC patients based on cervical microbiota and oral microbiota

**Fig. 3:**
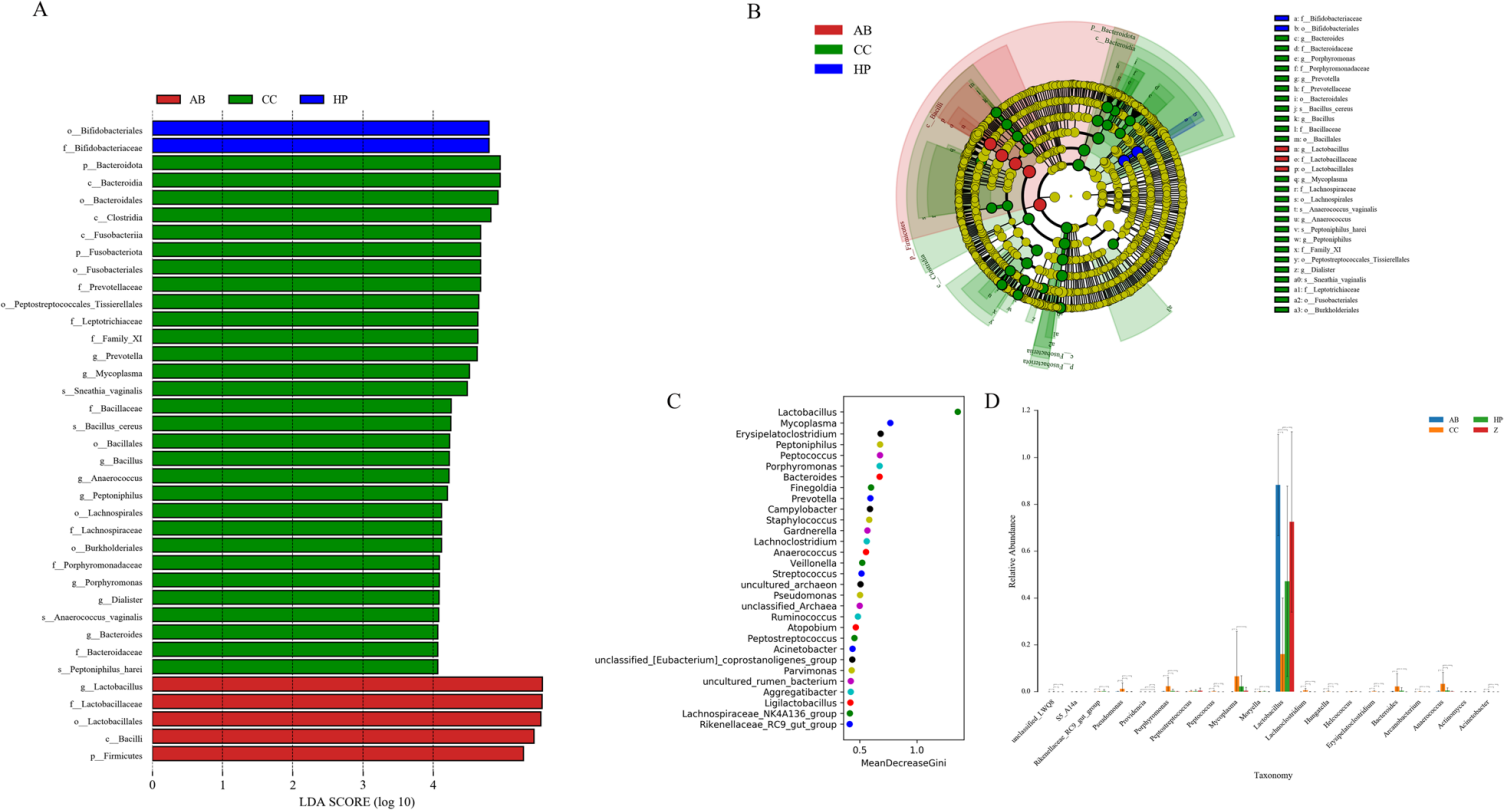
Identification biomarker in vaginal microbiome. Linear discriminant analysis (LDA) effect size (LEfSe) analysis among four groups. Clades in this graph were both statistically significant (*p* < 0.05) and had an LDA score > 4, considered a significant effect size. **A** Shows the representative genera; **B** Cladogram of tax associated with four groups. **C** Random forest analysis was used to screen the top 30 significant bacterial to distinguish CC, HP, AB groups and Z group; **D** Rank sum test screened the top 20 differential bacteria among four groups

A linear discriminant analysis (LDA) effect size (LEfSe) model was used to identify biomarkers in all groups, such as the Z, AB, HP and CC groups (*p* < 0.05, LDA > 2) (Additional file 1: Figure S2A). With an LDA score > 4, the most distinct vaginal biomarkers were identified (Fig. 3A, B). The CC group was enriched for genera including *Mycoplasma*, *Bacillus*, *Bacteroides*, *Dialister*, *Peptoniphilus*, *Porphyromonas*, *Anaerococcus*, *Prevotella*, and *Sneathia*. *Bifidobacteriales* was a biomarker in the HP group, while *Lactobacillus* predominated in the AB group. Random forest classification and rank sum testing verified the discriminatory species (top 30 and 20 bacterial species by abundance) (Fig. 3C, D). *Lactobacillus* was found to be the most important species for distinguishing the four groups, along with some other genera, similar to the LEfSe results. After performing tenfold cross-validation on random forest analysis between every two groups, several important species, including *Bacteroides*, *Hungatella*, *Lactobacillus*, *Ruminococcus*, *Moryella,* etc., were found to distinguish between the CC samples and the control samples. The receiver operating characteristic (ROC) curve effectively distinguished the CC group from the Z group (AUC = 93.75%) (Additional file 1: Figure S2D). The abundance of *Pseudomonas* differed between the HPV + and CC groups, with an area under the ROC curve of 87.5% (Additional file 1: Figure S2C, E). However, it was difficult to distinguish HPV-infected patients and controls (AUC = 64.29%) (Additional file 1: Figure S2F).

**Fig. 4:**
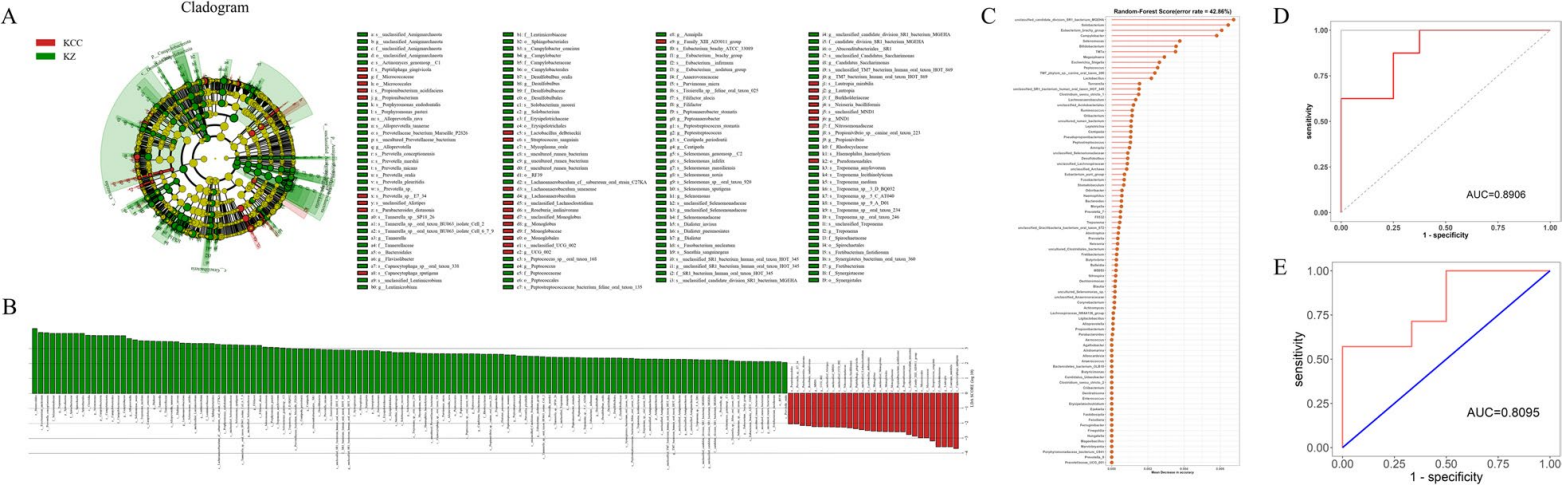
Oral biomarkers in patients with cervical cancer. **A** Shows the representative genera for KCC and KZ; **B** The signifcance taxa were tested by LEfSe analysis and showed using histogram. The threshold for the logarithmic LDA score was 2.0. **C** Random forest analysis with tenfold cross-validation was used to screen the different bacterial genera in the oral cavity of patients with cervical cancer. **D** Mean test prediction accuracy measured by the area under the ROC curve (AUC); **E** the receiver operating characteristic curves were calculated by combining LEfSe analysis and random forest analyses using the 25 genus-level signficant bacterium segregate cancer patients from controls

Subgingival microbiome LDA and LEfSe markers varied inversely to those of the vaginal microbiome, with control (KZZ group) plaques harbouring the most distinct taxa, including *Prevotella*, *Selenomonas*, *Saccharibacteria*, *Campylobacter*, *Amnipila*, and *Centipeda* (*p* < 0.05, LDA > 2; Additional file 1: Figure S3A, B). The markers of the AB group were *Pelospora* and *Filifactor*, and those of the HPV infection group were *Haemophilus* and *Gemmatimonadaceae*. *Monoglobus* was an effective marker of subgingival plaque in CC patients, but its relative abundance in the plaque microbiome was low. Combined with the previous diversity analysis, only two groups, KCC and KZ, were compared (Fig. 4A, B). We screened bacteria accounting for more than 10% of the total composition and found that *Capnocytophaga*, *Lautropia*, *Streptococcus*, *Lachnoanaerobaculum*, *Propionibacterium*, *F0332*, *Prevotella*, *Lactobacillus*, *Neisseria*, *Parabacteroides*, and *Roseburia* were oral markers for CC patients. The random forest model analysis was combined with tenfold cross-validation to construct the ROC curve, and the AUC reached 89.06% (Fig. 4C, D). The following bacteria, including *Fusobacterium*, *Campylobacter*, *Oribacterium*, *Selenomonas*, *Haemophilus*, *unclassified*_*SR1_bacterium_human_oral_taxon_HOT_345*, *Leptotrichia*, *Parvimonas*, *Phocaeicola*, *Capnocytophaga*, *Lautropia*, *Streptococcus*, *Lachnoanaerobaculum*, *Propionibacterium*, *F0332*, *Prevotella*, *Lactobacillus*, *Neisseria*, *Parabacteroides*, *Roseburia*, *Fretibacterium*, *Peptostreptococcus*, *All*o*prevotella*, *Pseudopropionibacterium*, and *Saccharibacteria,* were further screened as marker biomarkers, and although the AUC decreased to 80.95%, it had a certain accuracy.

### Association between microorganisms and environmental factors

**Fig. 5:**
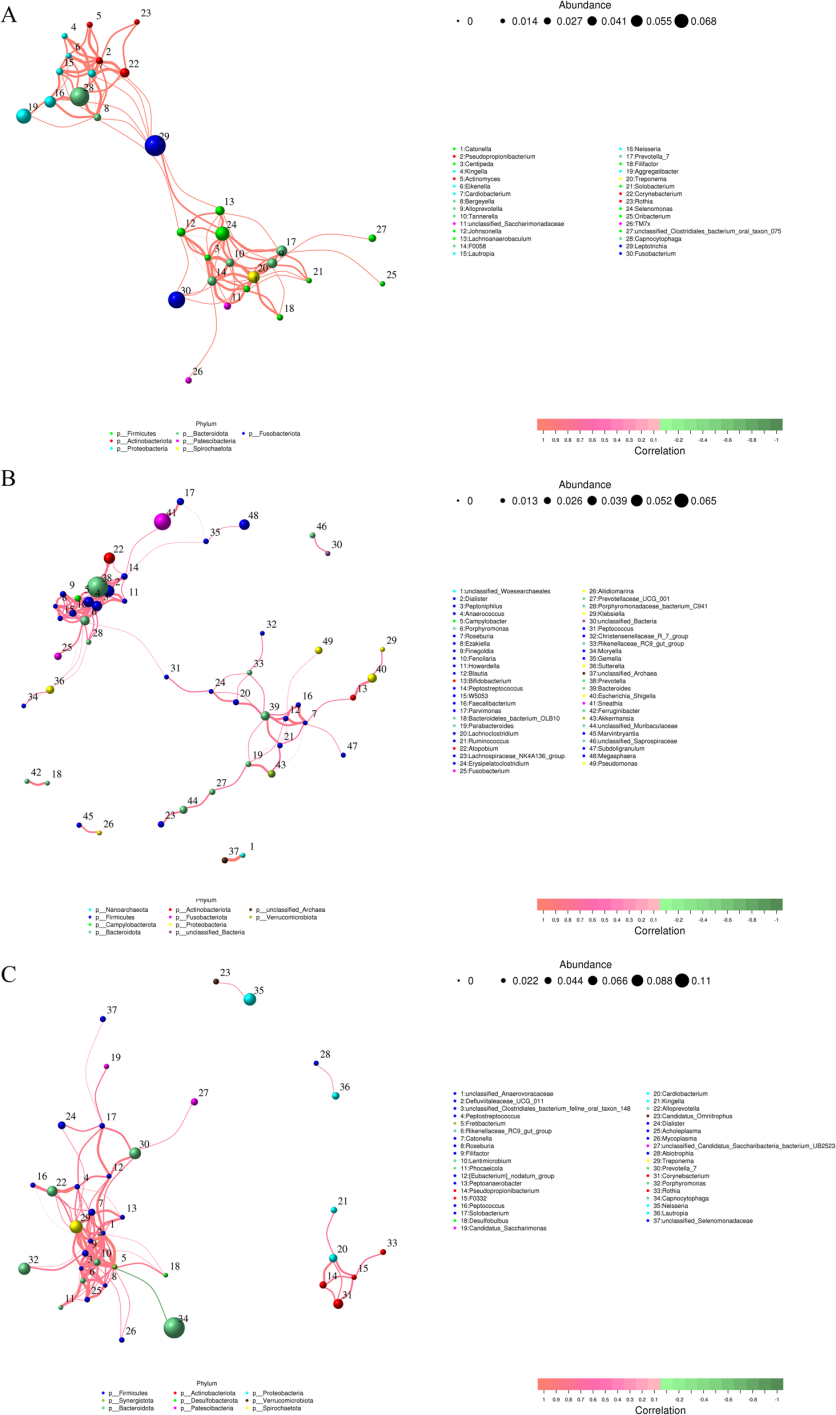
The correlation between bacteria. **A** Correlation network between different species in the vagina and oral. **B** The correlation at vaginal microbiota; **C** The correlation at oral microbiota

Co-occurrence networks revealed complexes of synergistically promoted microbes across vaginal and oral environments. The network showed the correlations between bacterial species common to both vaginal and oral microbiotas. The nodes represent bacterial species, and the edges (the lines connecting the nodes) represent the strength and type of correlation between these species. The node size likely corresponds to the abundance of the species, and the edge thickness might indicate the strength of the correlation (Fig. 5). The results showed that the presence of one species promoted the growth of another, indicating a synergistic effect. For example, *Treponema* and *Selenomonas*, *Lautropia* and *Capnocytophaga*, *Lachnoanaerobaculum* and *Leptotrichia*, *Lautropia* and *Neisseria*, *Saccharibacteria* and *F0058*, and *Tannerella* and *Fusobacterium* exhibited co-occurrence relationships (Fig. 5A). The correlations between the vaginal microbiota, such as *Peptoniphilus* and *Dialister*, *Porphyromonas* and *Peptoniphilus*, and *Porphyromonas* and *Dialister,* were also positive (Fig. 5B). However, there was a negative correlation between *Fretibacterium* and *Capnocytophaga* in the oral microenvironment, and other bacteria were positively correlated. In addition, *Peptostreptococcus* and *Roseburia* had multiple bacterial nodes (Fig. 5C).

**Fig. 6:**
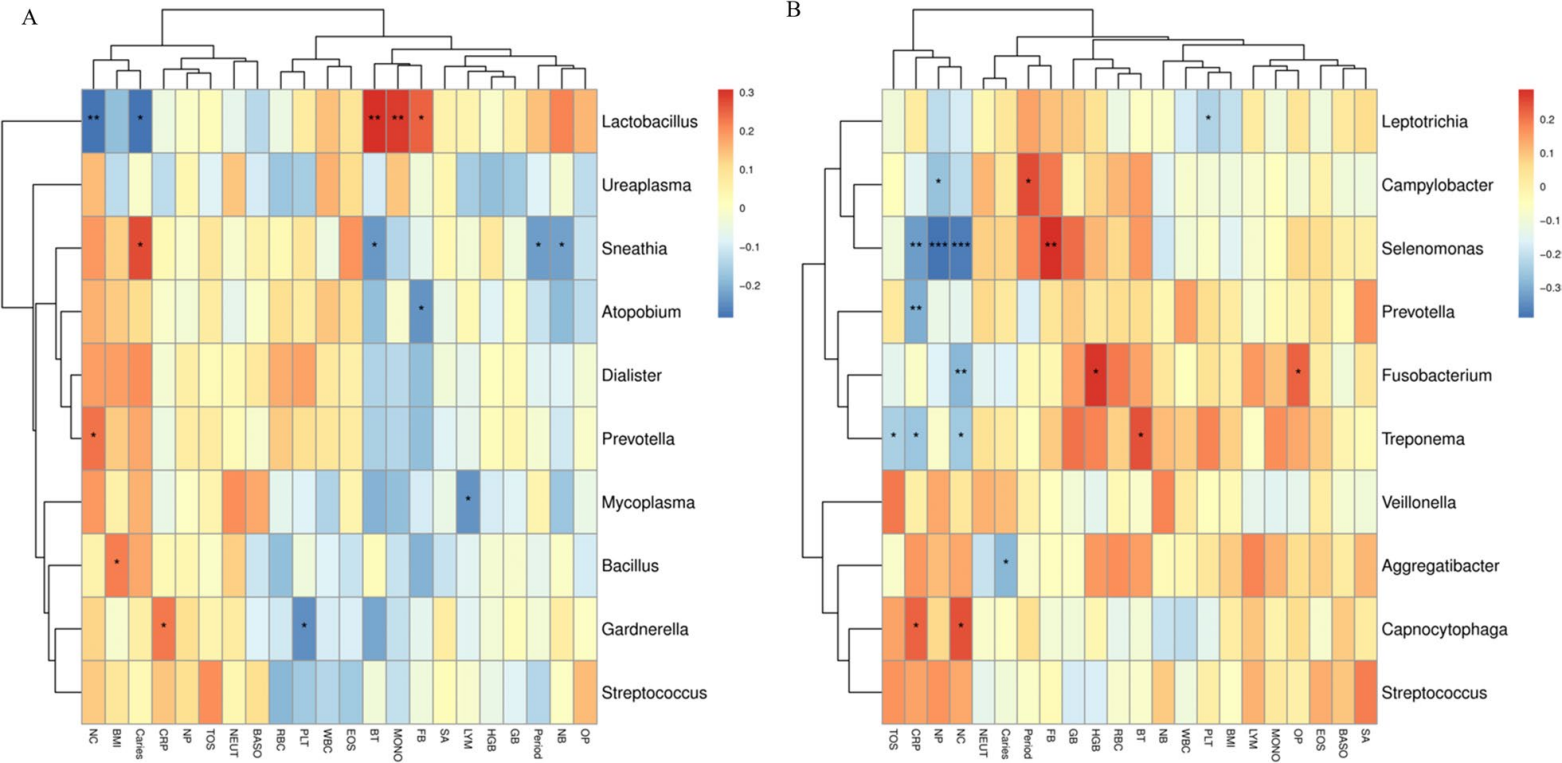
Correlations between microbiota species, demographic information, blood indices, oral disease and oral hygiene habits. **A** vaginal microbial community. **B** Oral microbial community. Spearman rank correlation coefficient is indicated using a color gradient, as follows: red indicates a positive correlation and blue indicates a negative correlation. *, *p* < 0.05; **, *p* < 0.01; and ***, *p* < 0.001. *SA* Sexual age, *BMI* body mass index, *NC* Number of childbirths, *NP* Number of pregnancies, *BASO* basophil percentage, *NEUT* neutrophilic granulocyte percentage, *EOS* eosinophils percentage, *MONO* monocytes percentage, *CRP* C-reactive protein, *LYM* lymphocytes percentage, *PLT* Platelets, *WBC* White Blood Cell Count, *RBC* Red Blood Count, *NB* Number of abortions, *HGB* hemoglobin, *GB* Gingival bleeding, *FB* Frequency of tooth brushing, *Period* Periodontitis, *Caries* Caries, *OP* Professional dental cleaning, *BT* Brushing time, *TOS*Toothache or sensitivity of tooth

We performed a Spearman analysis of these four groups to explore the correlations between the vaginal microbiome and demographic characteristics, blood indices, oral diseases and oral hygiene practices. Vaginal *Lactobacillus* abundance was negatively correlated with childbirth (*p* = 0.010) but was positively associated with Prevotella (*p* = 0.035) among the top ten most abundant vaginal species. BMI was positively correlated with the abundance of *Bacillus* (*p* = 0.046). In the vaginal microbiota, the abundance of *Lactobacillus* was positively correlated with the frequency of tooth brushing (FB) (*p* = 0.024) and inversely correlated with caries (*p* = 0.011). Similarly, the abundance of *Gardnerella* was positively related to the CRP level (*p* = 0.040) and was negatively correlated with the platelet (PLT) count (*p* = 0.026), brushing time (BT) (*p* = 0.005), and monocyte percentage (MONO) (*p* = 0.007). *Sneathia* abundance was positively correlated with caries (*p* = 0.012) but negatively related to BT (*p* = 0.035), number of abortions (NB) (*p* = 0.048), and periodontal disease (*p* = 0.039). The lymphocyte percentage (LYM) was negatively correlated with the presence of *Mycoplasma* (*p* = 0.031) (Fig. 6A).

For oral microorganisms, CRP was positively associated with the abundance of *Capnocytophaga* (*p* = 0.042) and negatively associated with the abundances of *Selenomonas* (*p* = 0.003), Prevotella (*p* = 0.007), and *Treponema* (*p* = 0.020). The number of births was positively correlated with the abundance of *Capnocytophaga* (*p* = 0.027) but negatively correlated with the abundances of *Selenomonas* (*p* = 0.001), *Fusobacterium* (*p* = 0.009), and *Treponema* (*p* = 0.025). Toothache or tooth sensitivity (TOS) was negatively related to the abundance of *Treponema* (*p* = 0.035). The abundances of the genera Campylobacter (*p* = 0.015) and *Selenomonas* (*p* < 0.001) were also negatively correlated with the number of pregnancies (NP). In addition, we observed a negative association between caries and the genus *Aggregatibacter* (*p* = 0.010), and periodontitis was positively associated with the genus *Campylobacter* (*p* = 0.022). FB and BT were significantly positively correlated with *Selenomonas* (*p* = 0.008) and *Treponema* (*p* = 0.027), respectively, and professional dental cleaning (OP) was strongly positively correlated with *Fusobacterium* (*p* = 0.045) (Fig. 6B).

### Predictive function analysis

**Fig. 7:**
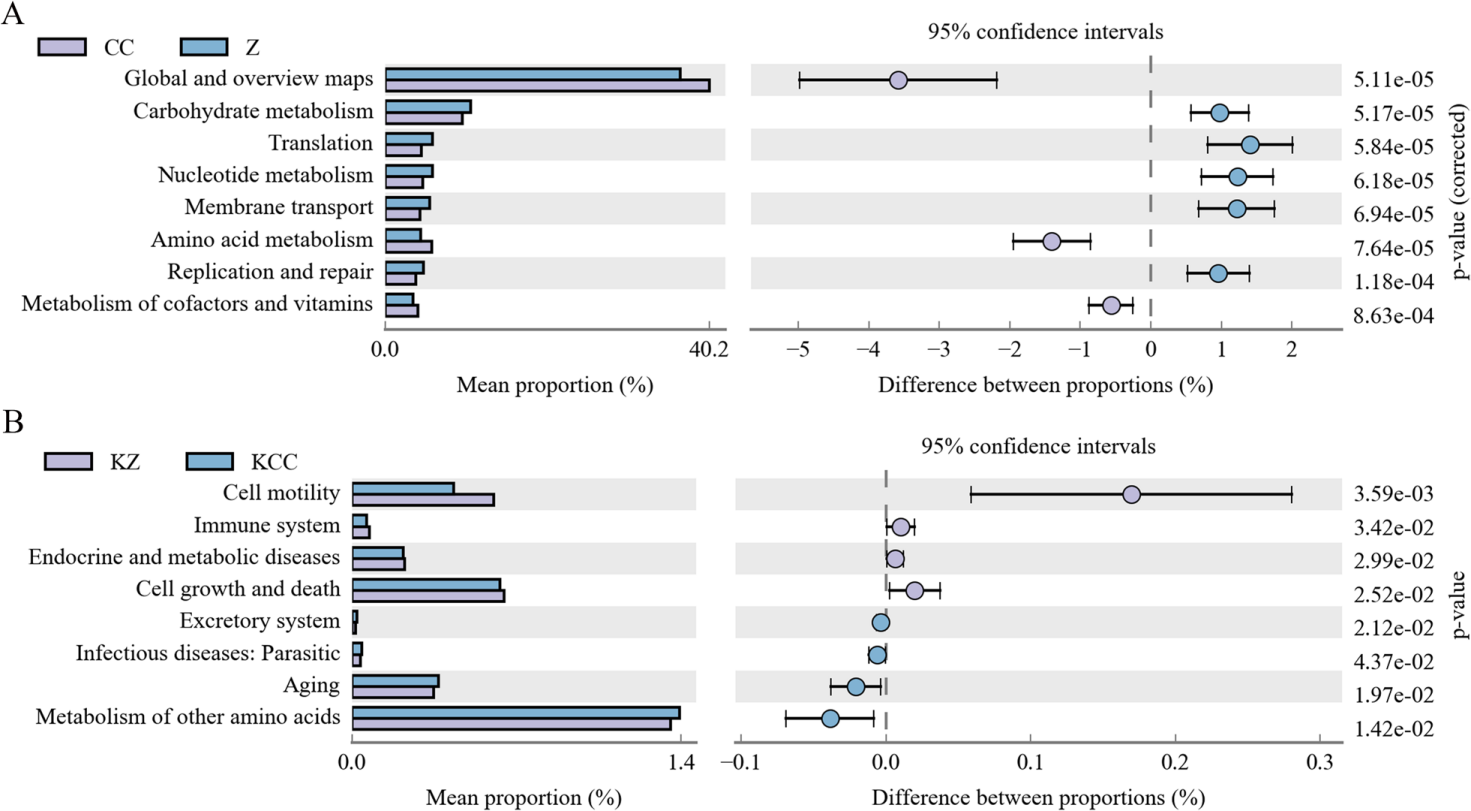
Predicted functions of the bacterial communities at the second level in different group. The middle shows the difference ratio of the function abundance within the 95% confidence interval, and the rightmost value is the p value. **A** KEGG pathway difference between CC group and Z group; **B** KEGG pathway difference between KCC group and Z group

Metagenomic functional potential based on 16S profiles revealed altered vaginal microbiome pathway activities that differentiated CC patients from controls (Z) (Fig. 7). The level 2 KEGG pathway analysis indicated that microbial gene functions, namely, global and overview maps, amino acid metabolism, and metabolism of cofactors and vitamins, were more abundant in the CC group than in the Z group. Pathways related to carbohydrate and nucleotide metabolism, translation, membrane transport, replication and repair were more abundant in the Z group (*p* < 0.05) (Fig. 7A). In addition, microbial gene functions related to metabolism of other amino acids, ageing, the excretory system, and infectious diseases were lower in the KZ group than in the KCC group. Pathways related to cell motility, cell growth and death, endocrine and metabolic diseases, and the immune system were more abundant in the KZ group than in the KCC group (*p* < 0.05) (Fig. 7B).

In addition, the CC group exhibited a greater abundance of genes related to amino acid metabolism than did the HP group and the AB group. The functional genes in metabolic pathways of CC patients were still more enriched in global and overview maps, amino acid metabolism, and metabolism of cofactors and vitamins, and those in the HP group were more enriched in global and overview maps and amino acid metabolism than those in the AB group (Additional file 1: Figure S4). This suggests that the presence of certain bacteria may play a role in the metabolic processes of cancer patients. There was a significant difference in the oral microbial flora structure between the KCC group and the KZ group, while in comparison with the other groups, there were similar differences. However, due to the small sample size, these findings should be interpreted with caution.

## Discussions

In this study, the composition and changes in the vaginal and oral microbiotas of women who experienced abortion, HPV infection or cervical cancer were evaluated. In addition to the different vaginal microecological environments of the four groups, we determined that there were significant differences in the composition, abundance, diversity, marker genera, and functional pathways of oral microorganisms between CC patients and healthy controls, which further proves that vaginal and oral microbes are not independent entities and that there is flora transfer between different parts of the body, which may be associated with systemic metabolism [22]. To our knowledge, this is the first study to explore the role of the oral microbiome in patients with CC, improving the accuracy of the CC screening process and broadening the scope of universal screening through insights gained from changes in the oral microbiome. Our team previously reported a meta-analysis and systematic review of CC specimens, which included cervical, vaginal, rectal, faecal, and urine samples without oral subgingival plaque (unpublished).

In exploring vaginal microecological shifts, there was no significant difference in the microbial composition between the AB group and the control group, which was dominated by *Lactobacillus* [23]. The effect of recurrent abortion on the vaginal flora is transient, regardless of cervical lesion type [24]. As long as surgery does not cause substantial damage to vaginal tissues, the equilibrium between commensal bacteria and opportunistic pathogens remains the same. With the occurrence of HPV infection and CC, the species diversity increased, the proportion of Lactobacillus gradually decreased, and the internal structure of the microbiome became more complex, similar to previous findings [25, 26]. The beta diversity of the Z group, AB group and HP group largely overlapped, which was significantly different from that of the CC group. It was further confirmed that the depletion of *Lactobacillus* and the increase in specific anaerobes (such as *Megasphaera*, *Prevotella* and *Gardnerella*) were related to cervical lesions [24]. LEfSe analysis identified cancer-specific vaginal biomarkers, including *Mycoplasma*, *Bacillus*, *Bacteroides*, *Dialister*, *Peptoniphilus*, *Porphyromonas*, *Anaerococcus*, *Prevotella*, and *Sneathia*, and the HPV + biomarker *Bifidobacterium* distinguished them from the control; the reliability of the experimental findings has been confirmed by other studies [13, 27–29]. *Bifidobacterium* is a beneficial microorganism of the intestinal flora that has many functions, such as resisting harmful bacteria, exerting antitumour, increasing immunity and improving gastrointestinal function, and it also exists in the oral cavity and vagina [30]. This research revealed that the use of *Bifidobacterium* species to distinguish cervical lesions is highly important for diagnosing women’s health conditions. The abundance of *Bifidobacterium* decreased, which is associated with high-grade squamous intraepithelial lesions (HSILs) [31, 32]. Wang et al. reported that the increased abundance of *Bifidobacterium* in the vaginal microbiome may be related to the clearance of HR-HPV infection, and focused ultrasound (FU) treatment may help to increase the abundance of *Bifidobacterium* [33], possibly because *Bifidobacterium* can survive in acidic environments and produce lactic acid and hydrogen peroxide, which have a protective effect on the vaginal environment [34].

Additionally, the shared periodontal pathogens *Porphyromonas* and *Prevotella* were identified as CC biomarkers, possibly because of the dynamic colonization of opportunistic bacterial pathogens on squamous epithelial cells in the oral or vaginal cavities and communication with the external environment. Oral pathogens can be transmitted from the gastrointestinal tract or through blood transmission to the vaginal cavity, and they can also be transmitted through person-to-person oral–genital contact [35]. This suggests that the oral cavity and vagina share a common microbial community and that there is also a reciprocal exchange of related microbial communities. The salivary microbiota of participants with bacterial vaginosis (BV) was more diverse than that of BV-negative participants [36], and *Prevotella intermedia* and *Porphyromonas endodontalis* were enriched in the subgingival gingival microbiota of BV-infected women compared to women without BV, which indicates the presence of a vagino–oral axis [37]. Moreover, *Porphyromonas* and *Prevotella* have been proven to have carcinogenic potential via several different mechanisms. For example, *Porphyromonas* can maintain chronic periodontal infection, leading to increased expression of proinflammatory molecules such as IL-6, IL-8, IL-1β, and TNF-α; activation of Toll-like receptors (TLRs) and antiapoptotic pathways (JAK/STAT and MAPK pathways); decreased expression of proapoptotic proteins; and increased cancer cell migration and invasion [38]. The gingipain protease *Porphyromonas gingivalis* activates NF-κB and MMP-9 in oral squamous cells, which are important for cancer cell invasion and metastasis [39]. Similarly, *Prevotella* produces virulence factors, fimbriae adhesins, lipopolysaccharides (LPSs), peptidoglycan and lipoteichoic acid, which induce the release of proinflammatory cytokines [40]. *Prevotella* can also stimulate tyrosine kinase receptors, degrade immunoglobulin, exert toxic effects on fibroblasts, and coordinate with other pathogens to promote the migration and invasion of cancer cells [41, 42]. Therefore, the pathogenic mechanisms of these periodontal pathogens are summarized as follows: they can stimulate chronic inflammation, inhibit cell apoptosis, activate cell proliferation and promote cell invasion, resulting in cancer [43].

Vaginal microbiome transformations along the cervical carcinogenesis route have been characterized, but the associated impacts on the oral niche remain underexplored. This study revealed that the oral cavity contains a significantly greater number of bacterial species than does the vagina and revealed extensive bidirectional sharing of microbial communities between the vaginal and oral cavities through an emerging vagino–oral axis [37]. This study provides insight into the microbial community shared between the oral and vaginal parts of the human body, as well as the exchange of related microbial communities. This study revealed that vaginal HPV infection and multiple abortions had no obvious impact on the oral flora of patients. However, the presence of CC can cause significant changes in the composition and abundance of oral microorganisms, leading to a lower diversity of the oral microbiome compared to that of the normal population, which is contrary to the changes in the vaginal microbiota. The prevalence of periodontal pathogens significantly increased in patients with CC. According to the results of both LEfSe and random forest analysis, *Fusobacterium*, *Campylobacter*, *Capnocytophaga*, *Veillonella*, *Streptococcus*, *Lachnoanaerobaculum*, *Propionibacterium*, *Prevotella*, *Lactobacillus* and *Neisser* were identified as oral bacterial markers for CC. Changes in the proportions of these bacteria can cause oral microflora dysbiosis and are also associated with all systemic diseases, including cancer [5, 38, 40, 44–47]. Different bacteria, such as *Fusobacterium nucleatum*, *Periodonticum*, *Streptococcus salivarius*, *Porphyromonas*, and different *Lactobacillus* subspecies, are associated with the diagnosis of this type of cancer. The periodontal pathogens *Fusobacterium nucleatum*, *Campylobacter*, *Pseudomonas aeruginosa* and *Porphyromonas* are considered “mobile microbiota” because they originate in the oral cavity but are also associated with extraoral infections and inflammation [45]. There are various tumorigenesis mechanisms associated with the oral microbiome, mainly including increased cell factors and inflammatory factors, chronic inflammation, cell proliferation, metabolic pathway changes, pathogenic bacterial metabolites, suppression of the immune response, induction of tumour genetic damage, and alteration of epithelial barriers [47–49]. Most of the current research focuses on how dysfunction of oral microorganisms affects major organs and systems of the whole body. However, there is a bidirectional relationship between oral and general health, and how systemic diseases adversely affect oral microorganisms needs further exploration.

In this study, a positive correlation was observed between vaginal and oral microorganisms, where certain bacteria exhibited connections with many others. Many studies have shown the synergistic effect of pathogenic microorganisms. Lo et al. reported that *Prevotella intermedia* is enriched in patients with CRC and enhances the migration and invasion of cancer cells; moreover, *Prevotella intermedia* and *Fusobacterium nucleatum* collectively contribute to the malignant transformation of colorectal adenoma into carcinoma [41]. *Streptococcus gordonii*, *Fusobacterium nucleatum* and *Porphyromonas gingivalis* synergistically promote the formation and proliferation of plaque biofilms, inhibit the growth of dendritic cells [50], and cause peripheral blood infection via bacterial tyrosine (BY) kinase (Ptk1), which is an important part of the signalling pathway that controls the synergistic interaction between *Porphyromonas gingivalis* and *Streptococcus gordonii* [51]. The colonization of vaginal epithelial cells by *Atopobium* enhances the virulence of *Gardnerella*, and biofilms formed by *Gardnerella* also dominate other bacteria for colonization [52]. The interactions of a variety of microbial synergy mechanisms are very complex. These mechanisms include not only metabolite cross-feeding but also a large number of microbiota quorum-sensing signals that result in enhanced resistance against the immune system, antibiotics, or direct contact between microorganisms to promote synergy [53]. Thus, the comprehensive impact of two or more microorganisms on disease is more severe than that of a single microorganism, and the complex interaction network may enhance the pathogenicity of multiple microbial infections, ultimately affecting disease initiation and progression. Notably, these studies are based on different bacteria at the same site, while the composition of the vaginal microbiome is different from that of the oral microbiome, and more studies are needed to clarify the mechanisms of action of the microbiome at different sites.

Systemic inflammation (such as cervical lesions and HPV infection) caused significant variation in the CRP concentration across all the groups. An increase in the number of pregnancies, childbirths and abortions can easily disturb the vaginal microenvironment, decrease the abundance of *Lactobacillus*, and increase the proportions of the anaerobic bacteria *Prevotella* and *Gardnerella*. Severe systemic and local inflammation are closely related to imbalances in the vaginal microbiome [54]. Similarly, when oral hygiene was good and brushing frequency was high, the abundance of pathogenic microorganisms associated with periodontal disease, such as *Selenomonas*, *Prevotella*, *Treponema*, and *Aggregatibacter*, decreased; this is due to oral microorganisms or their metabolites directly migrating through the blood or indirectly affecting the inflammatory mediators produced in the oral cavity [55].

Changes in microbial diversity in CC patients are consistent with changes in amino acid metabolism. Cancer cells grow rapidly, metabolites are overexpressed, and glycogen is consumed to produce large amounts of energy and intermediates [56]. It has been verified by metabolomics and transcriptomics that the occurrence of CC is significantly related to metabolism, proteolysis or proteoglycans [57]. With increasing cervical lesion severity, the consumption of lactic acid also gradually peaks, metabolic characteristics increase the consumption of glutamine after the development of CC, and these metabolite changes are negatively correlated with the abundance of *Lactobacillus* [58]. Studies in humans have shown that CC has distinct metabolic fingerprints in blood, tumour tissue, faeces, and urine [59]. However, metabolic maps of the oral cavity are lacking, and the PICRUST predictive analysis of this study provides additional data. Cervical lesions also affect oral microbial metabolites, and the amino acid metabolism of oral bacteria in patients with CC is greater than that in controls because of the presence of the corresponding pathogenic microorganisms. *Capnocytophaga* grows in places with high glycogen consumption [60], and *Porphyromonas*, *Prevotella* and *Fusobacterium* are closely related to differences in metabolites. Pyrimidine metabolism is significantly increased in patients with CC and is positively correlated with periodontal disease [61]. Glutamate, histidine, and tyrosine metabolism are also involved in the development of periodontitis. Cervical lesions lead to changes in the oral microenvironment, which may increase the pathogenic effect of periodontal bacteria, induce the selective growth and reproduction of oral pathogens, produce a more pathogenic microbiome, and even produce the “Warburg effect”.

## Limitations

As a case‒control study, we cannot conclude whether oral microbiome changes affect the vaginal microbiome or whether changes in the vaginal microbiome affect the oral microenvironment after the onset of cervical lesions, and the sample size is small. In the future, larger prospective cohort studies are needed to conduct long-term follow-up to further observe the relationship in order to confirm our findings. Second, CC patients were not tested for the presence of the HPV virus in the oral cavity. It is generally believed that oral sex may be linked to transmission from vaginal to oral sites [62], which causes viral infection of the oral mucosa and dysbiosis. However, the overall risk of oral HPV infection in published studies was low, and HPV transmission to the oropharynx by autoinoculation or oral-genital contact constitutes a rare and unlikely event [63]. Therefore, it is more likely that cervical lesions affect the oral microenvironment through retrograde flow. Third, the use of PICRUSt to predict microbiome function has some limitations, including the accuracy of functional prediction differences, and caution should be taken when interpreting these results. In addition, metagenomics or multi-omics combined analysis should also be utilized to accurately identify differences in gene function and metabolism.

## Conclusions

This study revealed changes in the vaginal and oral microbiota composition of women who underwent abortion, had HPV infection or developed CC. Decreased abundance of *Lactobacillus* and increased microbiota diversity (such as *Megasphaera*, *Prevotella* and *Gardnerella vaginalis*) were associated with progression from control through precancer to malignancy within the vaginal niche. HPV infection and CC not only have a certain impact on the vaginal microenvironment but also affect the oral microenvironment through synergistic effects on the microbiome and systemic metabolism. Pathways related to global and overview maps, as well as amino acid metabolism and the metabolism of cofactors and vitamins, are highly abundant in CC patients, exhibiting fluctuations in the microbiota. This can lead to a decrease in oral microbial diversity and an increase in the composition of pathogenic microorganisms, increasing the risk of infections and cancer. In the future, it is expected to screen cervical cancer through oral flora markers.

### Supplementary Information

**Additional file 1: Figure S1.** Microbial composition. Venn diagrams illustrating the number of bacterial differentially expressed OTUs from four groups, (**A**) vagina specimen; (**B**) subgingival plaque specimen. (**C**) Relative abundance of the cervical microbiota at the genus level; (**D**) Relative abundance of the oral microbiota at the genus level.

**Additional file 2: Figure S2.** Taxonomic differences at vaginal microbiome. (**A**) Linear discriminative analysis (LDA) effect size (LEfSe) analysis among four groups. (**B**) tenfold cross-validation on random forest analysis to distinguish between cervical cancer and normal groups of bacteria. (**C**) A random forest model to distinguish bacterial genera in patients with HPV infection and cervical cancer. Mean test prediction accuracy measured by the area under the ROC curve (AUC), (**D**) There was a high accuracy of distinguishment CC group (AUC = 93.75%) from Z group, (**E**) it distinguished patients with HPV infection from cervical cancer with an area under the ROC curve of 87.5%, (**F**) and it was difficult to classify HPV-infected patients and healthy people (AUC = 64.29%).

**Additional file 3: Figure S3.** Identification biomarker in oral microbiome. (**A**) LEfSe identifies bacterial clades that are differentially abundant within four groups. The threshold for the logarithmic LDA score was 2.0. (**B**) The signifcance taxa were tested by LEfSe analysis and showed using histogram.

**Additional file 4: Figure S4.** PICRUSt infers the cellular functions of bacterial communities in different groups. (**A**) AB group and CC group; (**B**) CC group and HP group; (**C**) AB group and HP group.

## Data Availability

The datasets presented in this study can be found in online repositories. The names of the repository/repositories and accession number(s) can be found below: https://www.ncbi.nlm.nih.gov/sra/PRJNA1055682.
