# Supplementary figures and images for: Relationship between vaginal and oral microbiome in patients of human papillomavirus (HPV) infection and cervical cancer
Source: J Transl Med. 2024 Apr 29;22:396. doi: 10.1186/s12967-024-05124-8 (PMC11059664; doi:10.1186/s12967-024-05124-8)

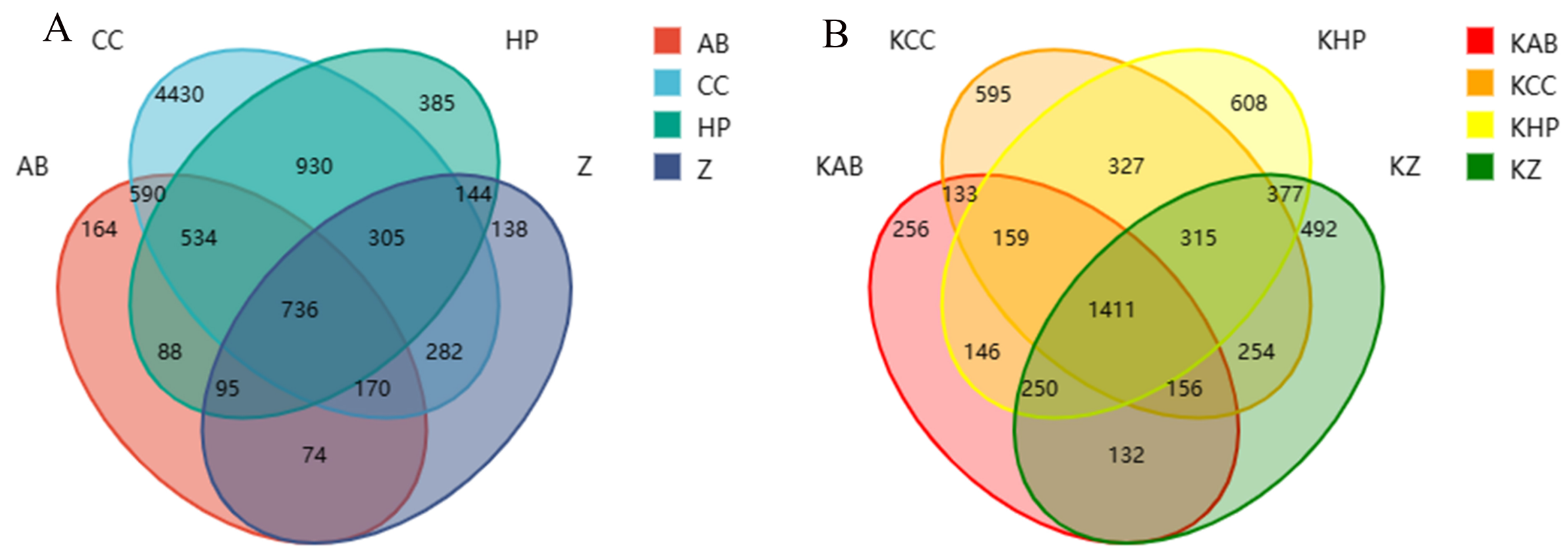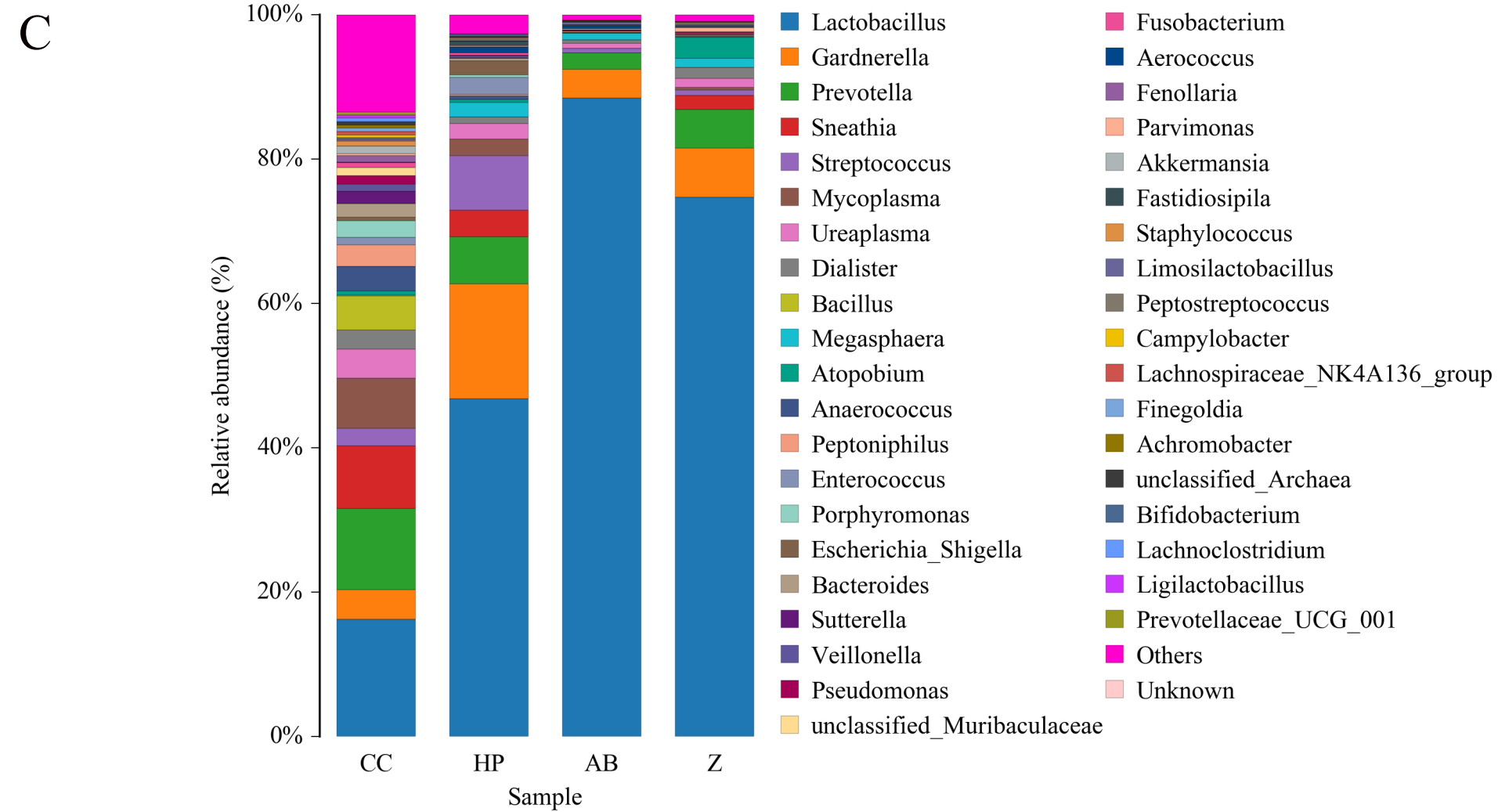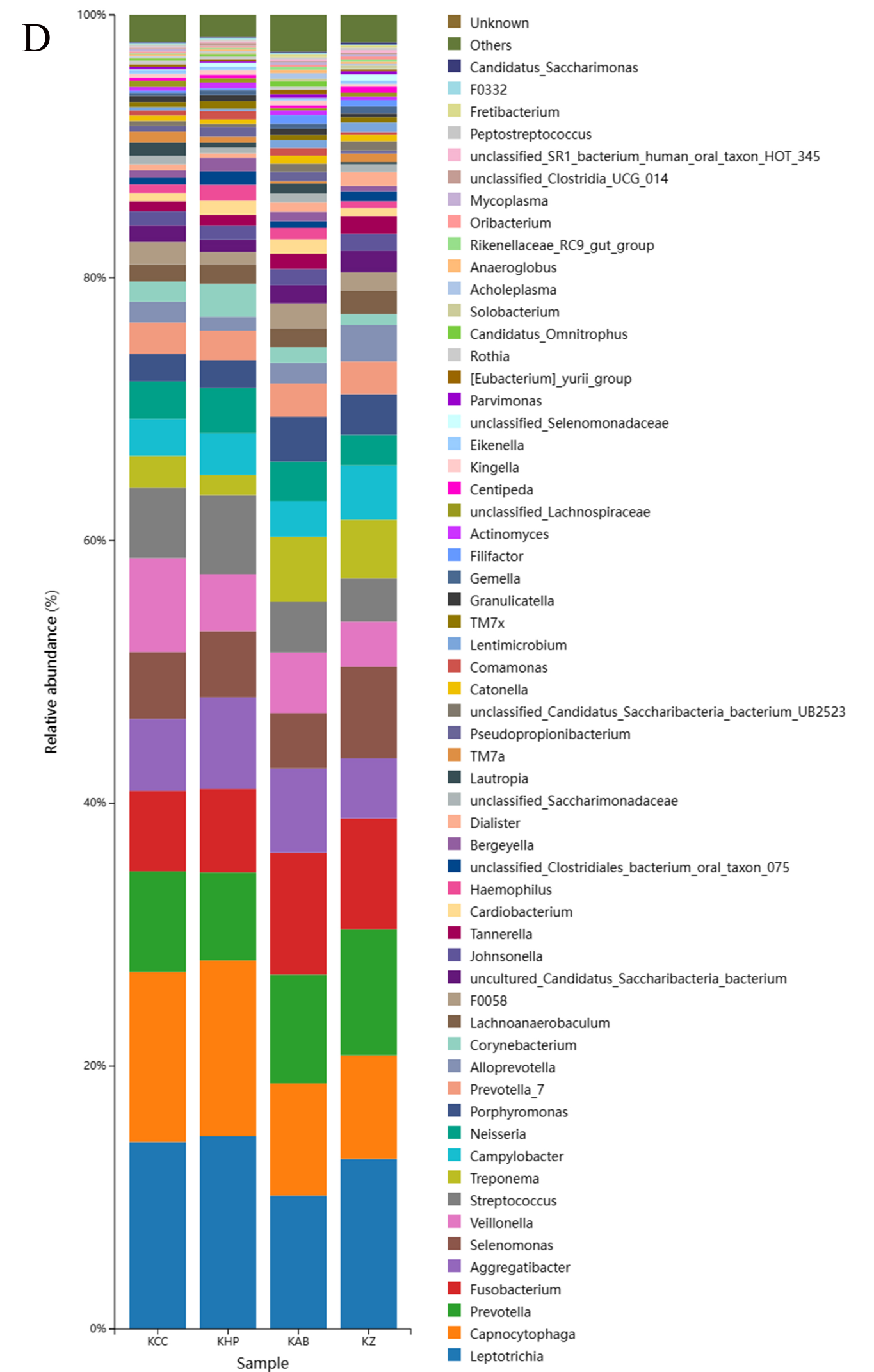

Supplement: Supplementary file 1 — Additional file 1: Figure S1. Microbial composition. Venn diagrams illustrating the number of bacterial differentially expressed OTUs from four groups, (A) vagina specimen; (B) subgingival plaque specimen. (C) Relative abundance of the cervical microbiota at the genus level; (D) Relative abundance of the oral microbiota at the genus level. [file 12967_2024_5124_MOESM1_ESM.pdf]

Cladogram

A

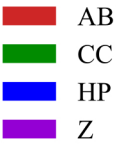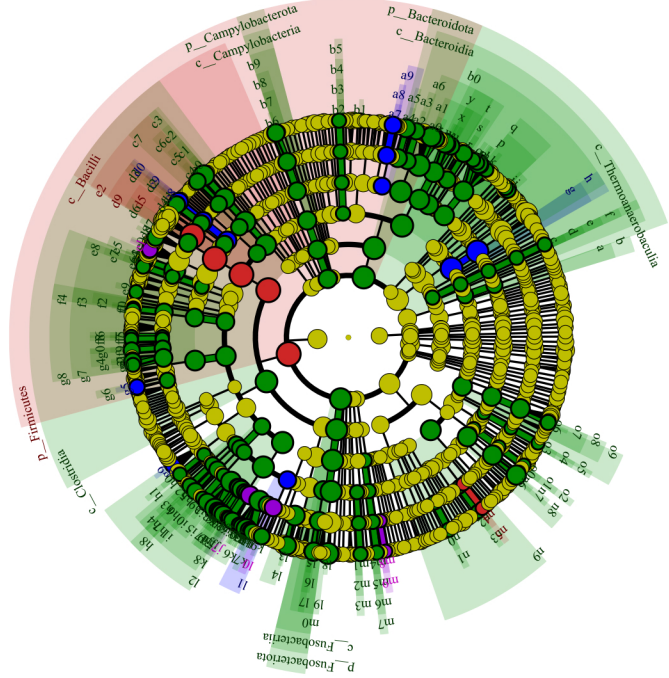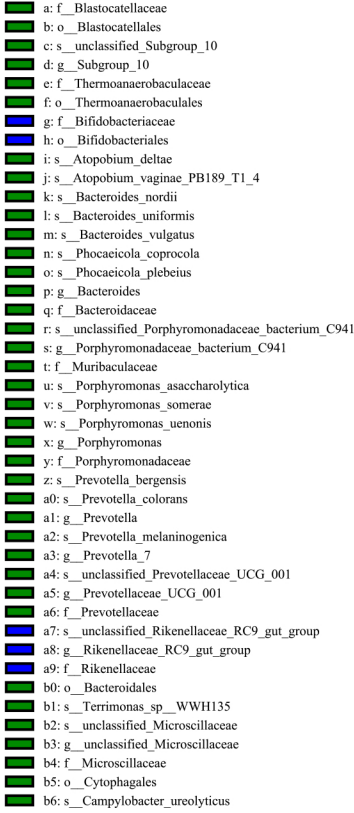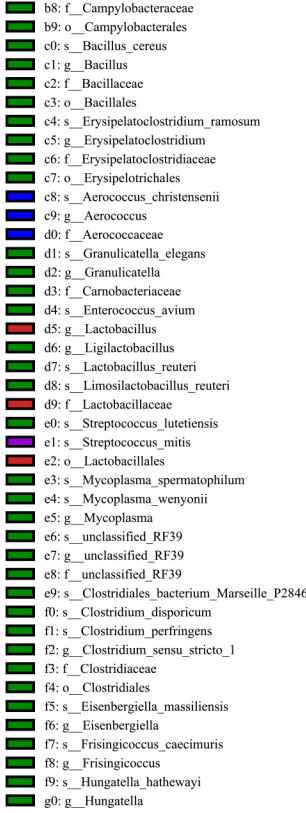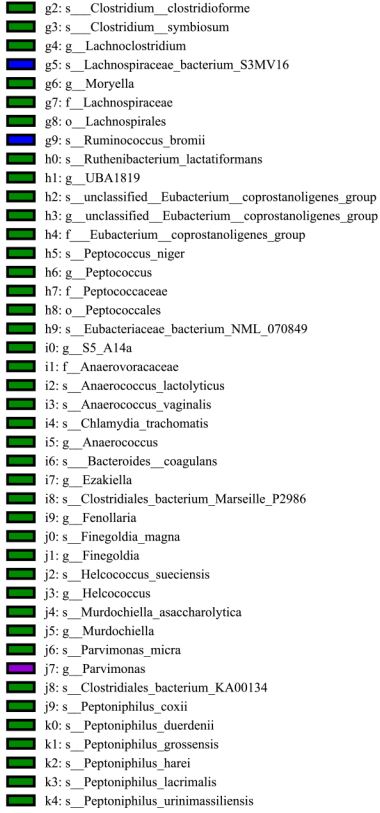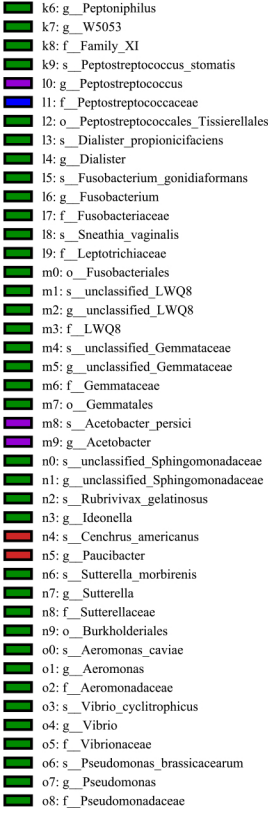

B

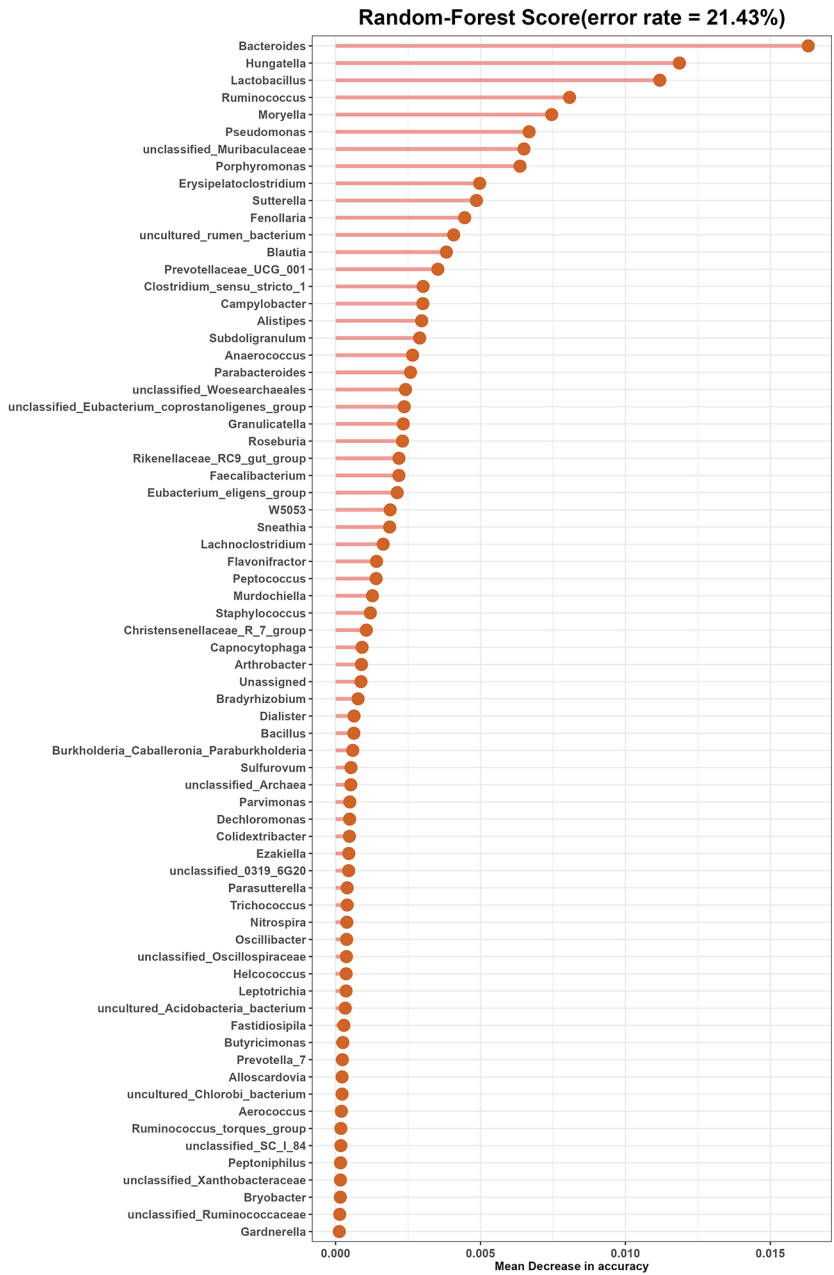

C

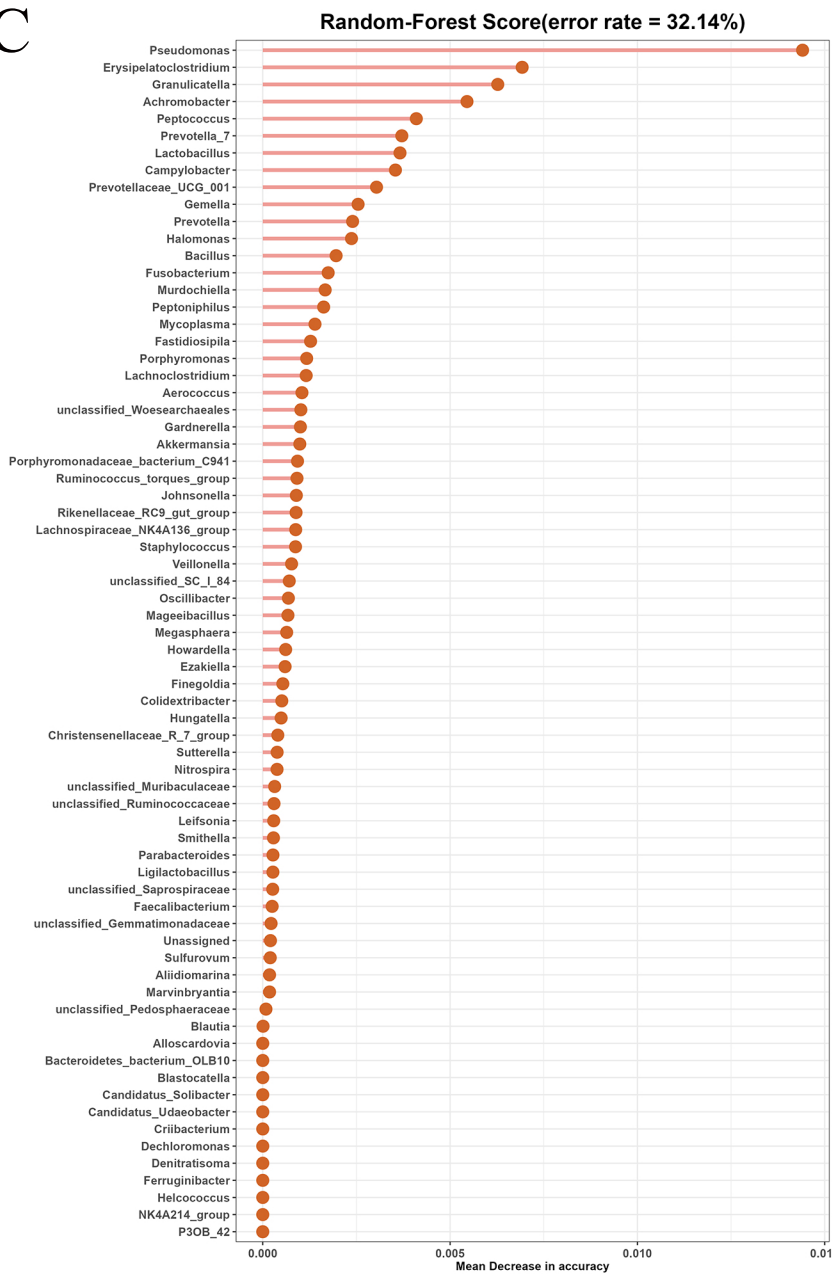

D

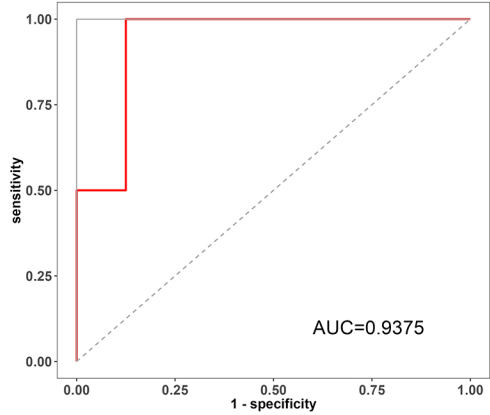

E

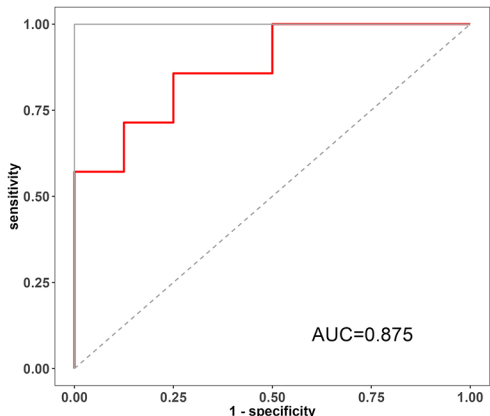

F

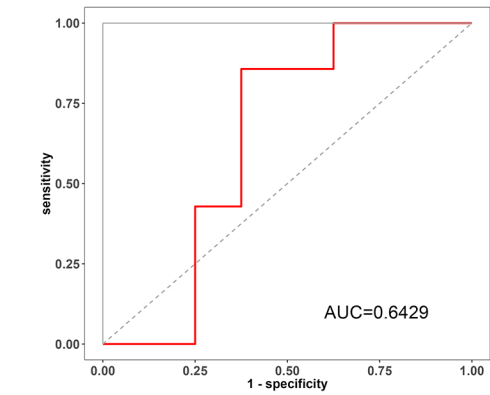

Supplement: Supplementary file 2 — Additional file 2: Figure S2. Taxonomic differences at vaginal microbiome. (A) Linear discriminative analysis (LDA) effect size (LEfSe) analysis among four groups. (B) tenfold cross-validation on random forest analysis to distinguish between cervical cancer and normal groups of bacteria. (C) A random forest model to distinguish bacterial genera in patients with HPV infection and cervical cancer. Mean test prediction accuracy measured by the area under the ROC curve (AUC), (D) There was a high accuracy of distinguishment CC group (AUC = 93.75%) from Z group, (E) it distinguished patients with HPV infection from cervical cancer with an area under the ROC curve of 87.5%, (F) and it was difficult to classify HPV-infected patients and healthy people (AUC = 64.29%). [file 12967_2024_5124_MOESM2_ESM.pdf]

A

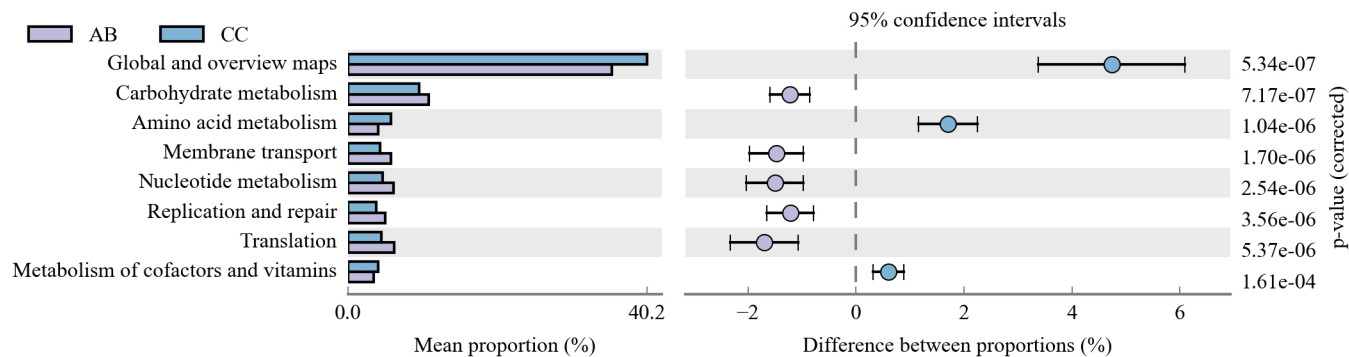

B

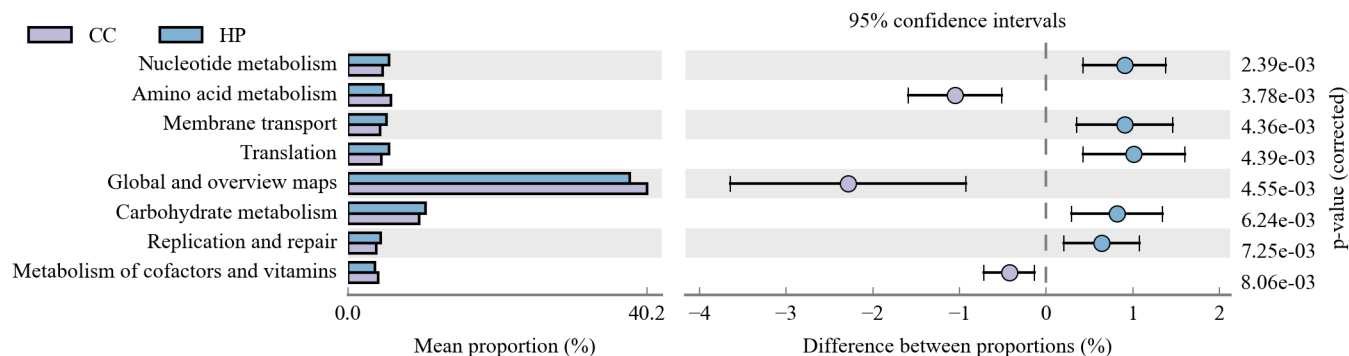

C

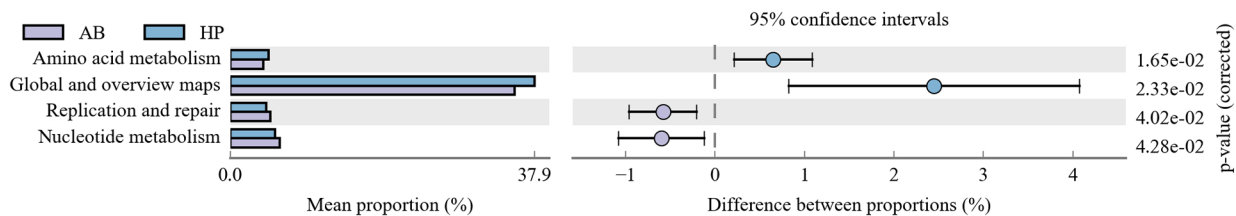

Supplement: Supplementary file 4 — Additional file 4: Figure S4. PICRUSt infers the cellular functions of bacterial communities in different groups. (A) AB group and CC group; (B) CC group and HP group; (C) AB group and HP group. [file 12967_2024_5124_MOESM4_ESM.pdf]
