# Supplementary material for: Relationship between vaginal and oral microbiome in patients of human papillomavirus (HPV) infection and cervical cancer
Source: J Transl Med. 2024 Apr 29;22:396. doi: 10.1186/s12967-024-05124-8 (PMC11059664; doi:10.1186/s12967-024-05124-8)
Supplement: Supplementary file 3 — Additional file 3: Figure S3. Identification biomarker in oral microbiome. (A) LEfSe identifies bacterial clades that are differentially abundant within four groups. The threshold for the logarithmic LDA score was 2.0. (B) The signifcance taxa were tested by LEfSe analysis and showed using histogram. [file 12967_2024_5124_MOESM3_ESM.pdf]

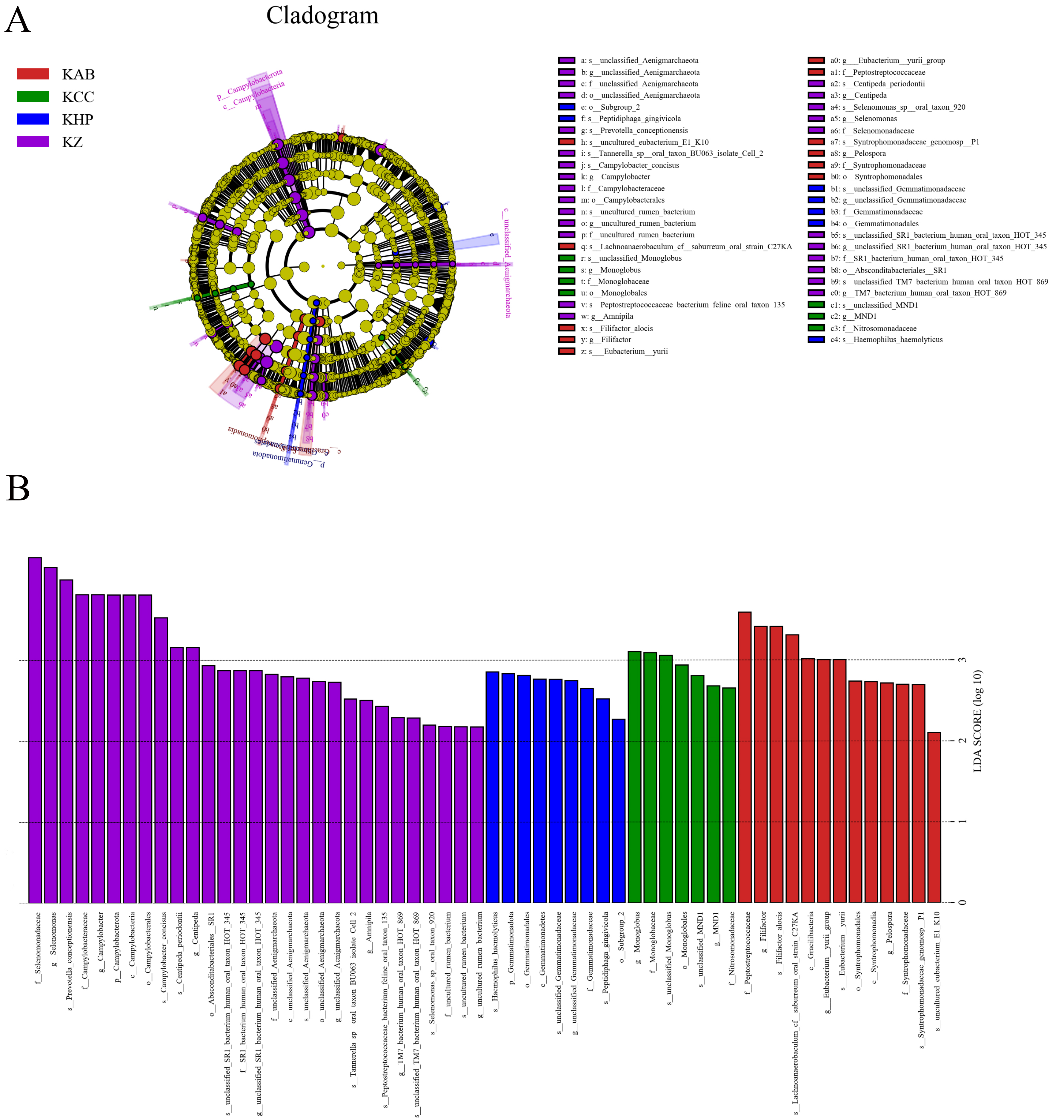

A
Cladogram
KAB
KCC
KHP
KZ
p__Campylobacterota
c__Campylobacteria
c__unclassified_Aenigmarchaeota
p__Gemmatimonadota
a: s__unclassified_Aenigmarchaeota
b: g__unclassified_Aenigmarchaeota
c: f__unclassified_Aenigmarchaeota
d: o__unclassified_Aenigmarchaeota
e: o__Subgroup_2
f: s__Peptidiphaga_gingivicola
g: s__Prevotella_conceptionensis
h: s__uncultured_eubacterium_E1_K10
i: s__Tannerella_sp__oral_taxon_BU063_isolate_Cell_2
j: s__Campylobacter_concisus
k: g__Campylobacter
l: f__Campylobacteraceae
m: o__Campylobacterales
n: s__uncultured_rumen_bacterium
o: g__uncultured_rumen_bacterium
p: f__uncultured_rumen_bacterium
q: s__Lachnoanaerobaculum_cf__saburreum_oral_strain_C27KA
r: s__unclassified_Monoglobus
s: g__Monoglobus
t: f__Monoglobaceae
u: o__Monoglobales
v: s__Peptostreptococcaceae_bacterium_feline_oral_taxon_135
w: g__Amnipila
x: s__Filifactor_alocis
y: g__Filifactor
z: s__Eubacterium__yurii
a0: g__Eubacterium__yurii_group
a1: f__Peptostreptococcaceae
a2: s__Centipeda_periodontii
a3: g__Centipeda
a4: s__Selenomonas_sp__oral_taxon_920
a5: g__Selenomonas
a6: f__Selenomonadaceae
a7: s__Syntrophomonadaceae_genomosp__P1
a8: g__Pelospora
a9: f__Syntrophomonadaceae
b0: o__Syntrophomonadales
b1: s__unclassified_Gemmatimonadaceae
b2: g__unclassified_Gemmatimonadaceae
b3: f__Gemmatimonadaceae
b4: o__Gemmatimonadales
b5: s__unclassified_SR1_bacterium_human_oral_taxon_HOT_345
b6: g__unclassified_SR1_bacterium_human_oral_taxon_HOT_345
b7: f__SR1_bacterium_human_oral_taxon_HOT_345
b8: o__Absconditabacteriales__SR1
b9: s__unclassified_TM7_bacterium_human_oral_taxon_HOT_869
c0: g__TM7_bacterium_human_oral_taxon_HOT_869
c1: s__unclassified_MND1
c2: g__MND1
c3: f__Nitrosomonadaceae
c4: s__Haemophilus_haemolyticus
B
LDA SCORE (log 10)
0
1
2
3
f__Selenomonadaceae
g__Selenomonas
s__Prevotella_conceptionensis
f__Campylobacteraceae
g__Campylobacter
p__Campylobacterota
c__Campylobacteria
o__Campylobacterales
s__Campylobacter_concisus
s__Centipeda_periodontii
g__Centipeda
o__Absconditabacteriales__SR1
s__unclassified_SR1_bacterium_human_oral_taxon_HOT_345
f__SR1_bacterium_human_oral_taxon_HOT_345
g__unclassified_SR1_bacterium_human_oral_taxon_HOT_345
f__unclassified_Aenigmarchaeota
c__unclassified_Aenigmarchaeota
s__unclassified_Aenigmarchaeota
o__unclassified_Aenigmarchaeota
g__unclassified_Aenigmarchaeota
s__Tannerella_sp__oral_taxon_BU063_isolate_Cell_2
g__Amnipila
s__Peptostreptococcaceae_bacterium_feline_oral_taxon_135
g__TM7_bacterium_human_oral_taxon_HOT_869
s__unclassified_TM7_bacterium_human_oral_taxon_HOT_869
s__Selenomonas_sp__oral_taxon_920
f__uncultured_rumen_bacterium
s__uncultured_rumen_bacterium
g__uncultured_rumen_bacterium
s__Haemophilus_haemolyticus
p__Gemmatimonadota
o__Gemmatimonadales
c__Gemmatimonadetes
s__unclassified_Gemmatimonadaceae
g__unclassified_Gemmatimonadaceae
f__Gemmatimonadaceae
s__Peptidiphaga_gingivicola
o__Subgroup_2
g__Monoglobus
f__Monoglobaceae
s__unclassified_Monoglobus
o__Monoglobales
s__unclassified_MND1
g__MND1
f__Nitrosomonadaceae
f__Peptostreptococcaceae
g__Filifactor
s__Filifactor_alocis
s__Lachnoanaerobaculum_cf__saburreum_oral_strain_C27KA
c__Gracilibacteria
g__Eubacterium__yurii_group
s__Eubacterium__yurii
o__Syntrophomonadales
c__Syntrophomonadia
g__Pelospora
f__Syntrophomonadaceae
s__Syntrophomonadaceae_genomosp__P1
s__uncultured_eubacterium_E1_K10
